# Integrity of the Intestinal Barrier: The Involvement of Epithelial Cells and Microbiota—A Mutual Relationship

**DOI:** 10.3390/ani12020145

**Published:** 2022-01-08

**Authors:** Małgorzata Gieryńska, Lidia Szulc-Dąbrowska, Justyna Struzik, Matylda Barbara Mielcarska, Karolina Paulina Gregorczyk-Zboroch

**Affiliations:** Department of Preclinical Sciences, Institute of Veterinary Medicine, Warsaw University of Life Sciences-SGGW, 02-786 Warsaw, Poland; lidia_szulc_dabrowska@sggw.edu.pl (L.S.-D.); justyna_struzik@sggw.edu.pl (J.S.); matylda_mielcarska@sggw.edu.pl (M.B.M.); karolina_gregorczyk_zboroch@sggw.edu.pl (K.P.G.-Z.)

**Keywords:** gastrointestinal tract, epithelial cells, goblet cells, Paneth cells, microbiota

## Abstract

**Simple Summary:**

The gastrointestinal tract is a complex organization of various types of epithelial cells forming a single layer of the mucosal barrier, the host mucosal immune system, and microorganisms termed as gut microbiota inhabiting this area. The mucosal barrier, including physical and chemical factors, spatially segregates gut microbiota and the host immune system preventing the development of immune response directed towards non-pathogenic commensals and dietary antigens. However, for the maintenance of the integrity of the mucosal surfaces, cross-talk between epithelial cells and microbiota is required. The microbiome and the intestinal epithelium developed a complex dependence necessary for sustaining intestinal homeostasis. In this review, we highlight the role of specific epithelial cell subtypes and their role in barrier arrangement, the mechanisms employed by them to control intestinal microbiota as well as the mechanisms utilized by the microbiome to regulate intestinal epithelial function. This review will provide information regarding the development of inflammatory disorders dependent on the loss of intestinal barrier function and composition of the intestinal microbiota.

**Abstract:**

The gastrointestinal tract, which is constantly exposed to a multitude of stimuli, is considered responsible for maintaining the homeostasis of the host. It is inhabited by billions of microorganisms, the gut microbiota, which form a mutualistic relationship with the host. Although the microbiota is generally recognized as beneficial, at the same time, together with pathogens, they are a permanent threat to the host. Various populations of epithelial cells provide the first line of chemical and physical defense against external factors acting as the interface between luminal microorganisms and immunocompetent cells in *lamina propria*. In this review, we focus on some essential, innate mechanisms protecting mucosal integrity, thus responsible for maintaining intestine homeostasis. The characteristics of decisive cell populations involved in maintaining the barrier arrangement, based on mucus secretion, formation of intercellular junctions as well as production of antimicrobial peptides, responsible for shaping the gut microbiota, are presented. We emphasize the importance of cross-talk between gut microbiota and epithelial cells as a factor vital for the maintenance of the homeostasis of the GI tract. Finally, we discuss how the imbalance of these regulations leads to the compromised barrier integrity and dysbiosis considered to contribute to inflammatory disorders and metabolic diseases.

## 1. Introduction

The mucosal surfaces form one of the major barriers protecting the host against invasion and systemic dissemination of both pathogens and local microbiota. In humans, mucosal surfaces cover over 400 m^2^ and can be classified into two types based on their distinct characteristics: type I mucosal surfaces that include the gastrointestinal (GI) tract, respiratory and female upper reproductive systems, and type II mucosal surfaces that are found in the visual, mouth alimentary, and female lower reproductive systems [1]. The basic difference is that type I mucosal surfaces are covered with the simple columnar epithelium, major secretory antibodies are immunoglobulins A (sIgA), and polymeric immunoglobulin receptor (pIgR) is present, while stratified squamous (non-keratinized), epithelium occurs on type II mucosal surfaces, the dominant isotype is IgG instead of IgA, and pIgR is absent [1]. 

The GI tract, together with gut-associated lymphoid tissue (GALT), is a specific system that is constantly challenged with contradictory stimuli. This is a region where the organism encounters more antigens than any other site of the body. In addition to its functions in food intake, digestion, absorption of food-derived nutrients, water and electrolytes exchange, endocrine and paracrine hormones production, it has to discriminate immediately between invasive pathogens and harmless food antigens as well as microorganisms that form gut microbiota. For pathogens, the major portals of entry are skin and mucosal surfaces. Enteroinvasive pathogens trigger a strong protective immune response that shields the GI tract, and by that means the host, against the development of the disease and further dissemination of the pathogens. However, such immune response towards non-pathogenic microorganisms that inhabit the GI tract and delivered dietary antigens will be wasteful; moreover, it can provoke hypersensitivity reactions and inflammation, accountable for barrier damage, and may lead to inflammatory disorders. As a result, specific mechanisms responsible for the tolerance induction had to develop to maintain the integrity of mucosal surfaces. The GI tract evolved together with intestinal microorganisms, resulting in the development of immune tolerance, dependent on the lack of responsiveness to the gut microbiota, rather than immune ignorance. Currently, the gut microbiota is considered a biological barrier, protecting against colonization of the GI tract with pathogens. Accordingly, this mutual GI tract-microbiota partnership does not lead to the stimulation of the immune response, however, at the same time, the physical barrier provides the sensing and defense mechanisms ready to protect against invading infectious agents. This recognition requires microbial sensing by host cells, which is carried out by pattern recognition receptors (PRRs), including Toll-like receptors (TLRs), nucleotide-binding oligomerization domain [NOD]-like receptors (NLRs), C-type lectin receptors (CLR), retinoic acid-inducible gene I [RIG-I] receptors (RLRs), absence in melanoma 2 [AIM]-like receptors (ALRs), which recognize microbial-associated molecular patterns (MAMPs) that are molecular structures essential for microbial survival or damage-associated molecular patterns (DAMPs), released from host cells facing injury or molecular stress [2,3]. Activation of PRRs induces several intracellular signaling pathways resulting in the cytokines and chemokines secretion as well as the transcription of other genes important for initiating and controlling the immune response. Since bacteria are the major component of microbiota, the intracellular signaling pathway dependent on TLRs most often is triggered, however, the involvement of other PRRs also occurs. TLR signaling can be organized based on the adaptor proteins involved in the intracellular pathway: myeloid differentiation primary response gene-88 (MyD88), engaged by TLR1, TLR2, TLR4, TLR5, TLR6 and TLR10, and TIR-domain–containing adapter-inducing IFN-β (TRIF), employed by TLR3, TLR4, and TLR9. Studies conducted on mice with disrupted either specific TLR or MyD88 indicated that sensing and recognition of commensal and/or pathogenic bacteria, as well as DAMPs *via* these receptors, is critical, on one hand, to the enforcement of protective barrier, and on the other hand, for signaling induction that, subsequently, will lead to the stimulation of protective immune response [3,4]. 

Inflammatory bowel diseases (IBD) are progressive disorders of the GI tract, broadly classified as either ulcerative colitis (UC), Crohn’s disease (CD), or are IBD-unclassified (IBDU) when they share features of both UC and CD. The incidence and prevalence of IBD are increasing with time and in different regions around the world and the elucidation of their pathogenesis is paramount [5,6]. They are characterized by chronic inflammation of the intestinal barrier. CD can affect all parts of the GI tract, with the primary manifestation site of the terminal ileum, and is characterized by transmural inflammation and epithelioid granulomas. In turn, UC always begins in the rectum, and major manifestation occurs within the colon, with inflammation restricted to the mucosal and submucosal parts of the intestinal wall [7]. The definite etiology and pathogenesis of IBD remain unclear, although genetic predispositions, defects, and alterations of local and systemic immune responses, environmental factors, are considered as triggers for IBD development. Other factors, like the loss of intestinal barrier function and composition of the intestinal microbiota, contribute to the onset of intestinal inflammation that can lead to the development of IBD. However, it still has to be resolved, if dysbiosis can induce IBD or if IBD accounts for gut dysbiosis [5,8].

In this review, we will focus on some crucial, innate mechanisms protecting mucosal integrity and being responsible for maintaining intestine homeostasis. Additionally, the involvement and characteristics of some epithelial cell populations that are bricks, building the first line of defense, participate in transferring signals between the luminal environment and immunocompetent cells in *lamina propria*, will be presented. To our knowledge, the interplay between epithelial cells and the GI tract microbiota is one of the most important interactions required for maintaining the integrity of the intestinal barrier and has recently highly improved. We will concentrate on the significance of this cross-talk in the prevention of intestinal inflammation.

## 2. The Structure and Function of the Intestinal Epithelium

**Figure 1:**
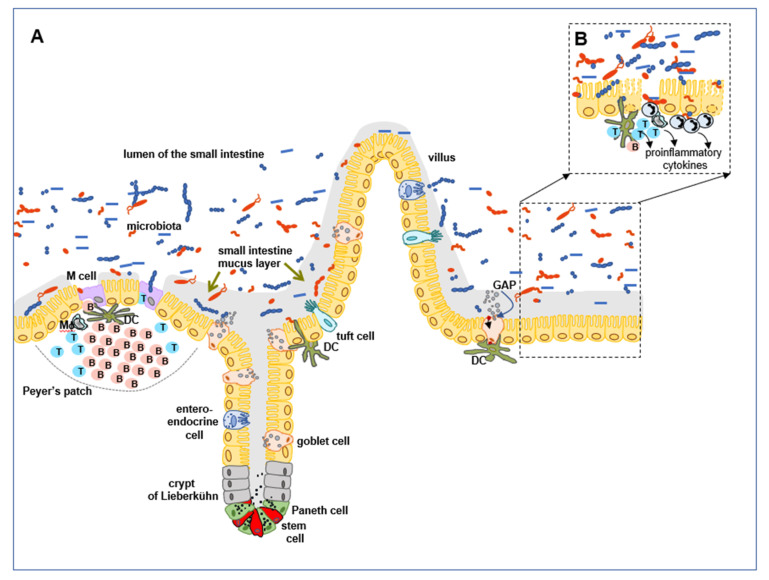
A representative schematic of villi, crypts of Lieberkühn and Peyer’s patches of the small intestine (according to [9]). (**A**) Constantly dividing stem cells, located at the base of the crypts of Lieberkühn, intercalating with Paneth cells, give rise to the progenitor cells differentiating into mature intestinal epithelial cells. Paneth cells, due to the proximity of stem cells and secretion of specific mediators, are considered to be protectors of this population. Moreover, they are the major source of antimicrobial peptides found in the mucus of the small intestine, and are responsible for controlling the gut microbiota, preventing its overgrowth, and inhibiting inflammation. Enterocytes, absorptive cells, comprise the most numerous populations, and together with goblet cells, are found in the crypt-villus axis, and follicle-associated epithelium (FAE). Goblet cells are responsible for the formation of the mucus layer covering the small intestine epithelium, due to the secretion of mucin MUC2. The mucus barrier in the small intestine is penetrable for microbiota. Goblet cells also facilitate luminal antigen sampling and their transport to the *lamina propria* located dendritic cells *via* goblet cell-associated passages (GAPs). FAE overlying Peyer’s patch, contain scattered, devoid of mucus layer M cells, which participate in direct sampling of luminal antigens, translocating them to the basolateral site where antigen presenting cells, located in *lamina propria*, can capture, process, and present them, inducing tolerance or antigen-specific immune response. Tuft cells and enteroendocrine cells are sparse in the intestinal lining. They are involved in protection against helminth infection or secrete peptides and hormones which stimulate, among others, intestinal peristalsis. (**B**) Disturbance of the mucus barrier results in penetration and direct contact with epithelial cells with microorganisms. This results in inflammatory response induction, recruitment of neutrophils, induction of dendritic cells and macrophages localized in *lamina propria*, stimulation of T cells and B cells, and secretion of proinflammatory cytokines. Inflammatory response leads to the injury of epithelial cells.

The physical barrier in the GI tract is formed by epithelial cells. Small and large intestines form a major part of the GI tract and the epithelial cells in these areas differ in their structure and composition. The small intestine consists of three sections: duodenum, jejunum, and ileum. Specific, finger-like projections, seen on the mucosal surface that protrude into the lumen, form villi, increase mucosal area and adsorption rate of the nutrients enzymatically digested in the duodenum. Additionally, invaginations of the small intestine mucosa occur, forming crypts of Lieberkühn, which are comprised, predominantly, of proliferating stem cells localized at the base of the crypts (Figure 1).

The large intestine (colon) that connects to the small intestine from one end and the anus at the other, comprises four parts: the cecum, colon, rectum, and anal canal. It is an important part of the digestive system and participates in reabsorbing water, absorbing some vitamins, and processing undigested food material (e.g., fiber). Colonic mucosa is devoid of villi, however, it contains deep tubular crypts that increase in depth towards the rectum. Absorptive, columnar cells, endocrine cells, goblet cells, and stem cells form colon epithelium. There are no Paneth cells. Epithelial cells are renewed every 6 days [10].

The gut epithelium is one of the most rapidly proliferating cells in mammals. To maintain epithelial integrity, those cells are continuously replaced by proliferating progenitors, derived from multipotent intestinal stem cells (ISCs), localized in the base of the crypts of Lieberkühn and colon crypts. Constantly dividing stem cells give rise to progenitors that differentiate into mature intestinal epithelial cells (IECs) that can be grouped by the functions, with newly formed epithelial cells migrating upwards toward the villus tip, with one exception. Newly formed Paneth cells, present only in the small intestine, move downwards further into the crypt where they mature and start to play their crucial role in maintaining homeostasis of the gut [11,12]. 

Epithelial cells can be distinguished by their ability of proliferation, the renewing rate, and age (Figure 1) [11]. The aged IECs undergo apoptosis and are shed off into the intestinal lumen. Meanwhile, Paneth cells escape from the crypt bottom by cellular fragmentation and are phagocytosed by macrophages infiltrating from *lamina propria* [13]. It is estimated that under homeostatic conditions the entire ileal crypt is replaced every 4–5 d [10,11,14].

Various differentiated cell populations in both, small intestine and colon, comprise (i) the most numerous population of enterocytes, named colonocytes in the large intestine, (ii) secretory lineages, such as goblet cells—source of the mucus, (iii) enteroendocrine cells, which secrete peptides and hormones (cholecystokinin, serotonin), which stimulate intestinal peristaltic movements that renew the mucus layer, (iv) tuft cells, participating in the clearance of parasites from intestinal lumen by synthesizing IL-25 and, subsequently, polarizing Th2 immune response, (v) Paneth cells in the small intestine or deep crypt secretory cells in the colon (vi) and microfold (M) cells, located within the follicle-associated epithelium (FAE), overlying Peyer’s patches and binding luminal antigens to transport them to the subepithelial regions, where they are captured and processed by dendritic cells (DCs), that afterwards migrate to mesenteric lymph nodes (MLNs), and stimulate immune response (Figure 1) [14,15,16,17]. It has to be noticed that M cells mainly located in the epithelium overlying Payer’s patches can also be found in isolated lymphoid follicles, appendix, colonic patches, and nasopharyngeal associated lymphoid tissue. They act as the entrance gate and deliver luminal antigen to the *lamina propria* localized immunocompetent cells in the small intestine. However, in the colon under inflammatory or infectious conditions, M cells are responsible for increased bacterial translocation and they enhance inflammatory response [18,19]. 

All these intestinal epithelial cell populations collaborate to form a protective barrier that confines gut microbiota in the intestinal lumen. At the same time, epithelial cells serve as a link to immunocompetent cells localized in *lamina propria*, the effector site of the GALT, sending information through direct cell-cell contact or mediators (cytokines and chemokines), thus priming immune response or immune tolerance. GALT includes multi-follicular lymphoid tissues, such as Peyer’s patches of the small intestine and numerous isolated lymphoid follicles that are distributed along the length of the small and large intestines. 

The specific structure of Peyer’s patches, located just below FAE, allows for the direct contact of antigens delivered to the basolateral area, with cells localized in the subepithelial dome (SED). SED is dominated by professional antigen presenting cells (APCs) such as DCs and macrophages, as well as diverse populations of T cells and B cells of IgA, IgG, and IgM isotypes. Bacteria that penetrated the epithelial barrier without triggering a stronger immune response are removed by resident macrophages, and upon inflammation other macrophage populations are recruited to this area. The interplay between DCs and epithelial cells is very important. DCs as the professional APCs, can interact with transported antigens or send their dendrites through the intercellular junctions of epithelial cells to the lumen, for sampling luminal antigens, and they also respond to secreted cytokines and chemokines. Subsequently, antigen-primed DCs interact with T cells residing in SED, and this interplay results in antigen-specific immune response stimulation, or T cell polarization towards tolerance induction. Of special importance are B cells that are activated in SED *via* their BCRs by M cells-delivered antigens, secrete IgA that transferred by transcytosis through enterocytes into the lumen, control intestinal microbiota and invading pathogens. These interactions occurring in *lamina propria* are bidirectional, and immunocompetent cells, through released mediators, influence the physiology of epithelial cells. The important factors are IL-10 and TGF-β that are produced by many cells, including CD4^+^ T cells, some populations of macrophages, and other cells located in *lamina propria*. These two cytokines act as anti-inflammatory factors limiting the expansion of effector cells, inducing proliferation of regulatory T cells, and maintaining immune tolerance [20,21,22,23]. Thanks to such an enormous complexity of IECs, their cooperation with GALT and with microbiota, the regulation and maintenance of the GI tract homeostasis, and immediate mobilization of defense against hazardous signals is possible.

## 3. Epithelial Cells—Their Involvement in the Formation of the GI Tract Protective Barrier

The major functions of epithelial cells in the GI tract are ‘segregation’ and ‘mediation’ to maintain the state of unresponsiveness towards microbiota. The term ‘segregation’ is simply the separation of microorganisms and/or their components in the gut lumen from the sterile, deep tissue. This separation is achieved, partially, by forming two types of barriers: physical and chemical, to spatially isolate luminal gut microbiota and immune cells localized in *lamina propria* and intercalate epithelial cells to prevent the development of inflammation. The physical barrier, commonly regarded as a monolithic wall-like structure, in fact, consists of numerous specialized components, heterogeneous cell types, and intercellular junctions. On the other hand, the term ‘mediation’ represents the delivery of signals from gut microbiota or pathogens to the host immune cells. Epithelial cells are able to react upon signals delivered by microorganisms or their metabolites, as well as signals delivered by gut immune cells through direct cell-cell contact or chemokines and cytokines. Secretion of such mediators results in mobilization of protective immune mechanisms or inducing oral immune tolerance. The physical barrier comprises of the mucus layer that covers the surface of the GI wall, the glycocalyx on the microvilli of absorptive enterocytes, and the cell junctions responsible for tight sealing epithelial cells, forming an obstacle impenetrable for microbiota and pathogens. The chemical barrier is made up of many constituents, like digestive acids secreted by the GI tract, digestive enzymes, mucopolysaccharides, glycoproteins, glycolipids, and other compounds defined as antimicrobial peptides (AMPs). The biological, physical as well as chemical barriers, form mutually dependent microecosystem that, together with GALT, are responsible for maintaining the GI tract homeostasis.

The first barrier in the GI tract is mucus. It coats the surface of the intestine forming an obstacle that protects against binding and infiltration of the epithelium not only with the intestinal microbiota but also with enteric infectious agents as well as digested food antigens and food-associated toxins. Continuously secreted mucus forms a protective layer, expresses a very important feature–viscosity, which is balanced to ensure entrapment of microbiota, pathogens, but at the same time enables the flow along the mucosal surfaces. This protective layer presents different properties in the stomach, small intestine, and colon. Mucus, a viscous fluid, is secreted by the goblet cells, named by their shape, featuring the ability to store and secrete granules into the lumen of the intestine. In the large intestine, where the density of microorganisms is much greater than in the small intestine, the number of goblet cells is much higher. 

### 3.1. Mucus Barrier Formation

Mucus is formed primarily of mucins, highly glycosylated large proteins characterized by the presence of 3 domains: the N’ terminal domain, central domain–composed of protein core (called PTS sequences), containing the amino acids residues: proline, threonine and serine, and C’ terminal domain. The protein core of mucin is protected from endogenous protease degradation, due to glycan formation dependent on O-glycosylation within the Golgi apparatus [20,24,25]. There are more than 20 subtypes of mucins identified in humans. They are either secreted or membrane bound-mucins, and their presence varies throughout the GI tract, for example, MUC6 is found in the duodenum in Brunner’s gland, MUC5B and MUC7 are produced by salivary glands, however, the major, best known and studied mucin found in the small and large intestine, is MUC2, which is built by monomers of about 2.5 MDa and 20% comprise the protein core while the rest is glycan [15,24,26,27,28,29].

Before secretion, MUC2 is stored in the goblet cell granules, where low pH, high Ca^2+^ concentration, and the absence of water, promote organized storage conditions. Upon release, it is necessary to expose mucins to an increased pH and to decreased calcium concentration. Therefore, the Ca^2+^ is chelated with bicarbonate ions (HCO_3_^−^), provided by the cystic fibrosis transmembrane conductance regulated channel, leading to alkalizing conditions and decreasing in Ca^2+^ concentration, compared to the environment in the mucus granules. In such conditions, the packed mucins unfold to organize spontaneously a sieve-like structure, which allows the passage of small molecules and impedes the migration of microorganisms from the gut lumen [24,30,31,32].

**Figure 2:**
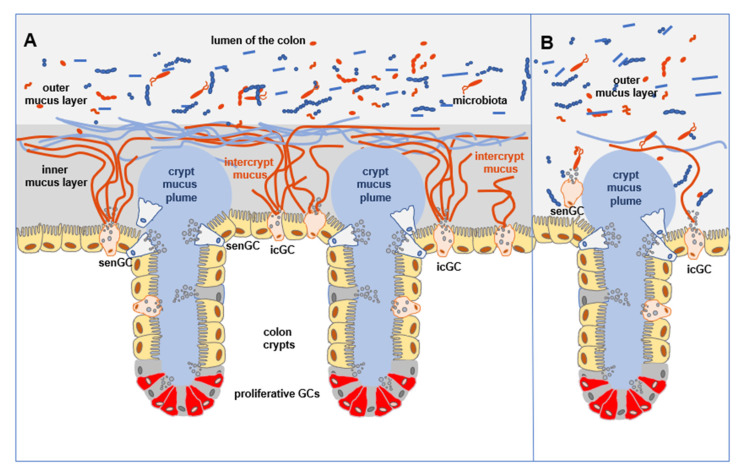
A representative schematic of colon crypts (according to [32]). (**A**) Colonocytes are the most numerous epithelial cell population of the colon. Constantly dividing stem cells differentiate into proliferative goblet cells that give rise to diverse populations of goblet cells that participate in the formation of mucus barrier, due to secretion of MUC2. The colon mucus barrier is formed of the inner mucus layer, free of microbiota and outer mucus layer, penetrable for microorganisms. Thick crypt mucus plumes, impermeable for bacteria, shield stem cells area, are secreted by crypt goblet cells among which, at the entrance to the crypt are located sentinel goblet cells (senGCs). Upon TLR ligand stimulation, senGCs form NLRP6 inflammasome, and the production of reactive oxygen species occurs. This will lead to the release of Ca^2+^ that passed through gap junctions to neighboring cells, inducing mucus release. Activated senGCs are expelled to the lumen. Between crypts lie highly differentiated intercrypt goblet cells (inGCs), secreting intercrypt mucus, penetrable for small molecules. Intercrypt mucus and crypt mucus plume form the colon inner mucus layer. Together, populations of goblet cells form a network that protects epithelium against microbial colonization. Any disruption of inGCs results in epithelial exposure to bacteria and the possible development of colitis. (**B**) The diminished number of inGCs due to the increased cell shedding to the lumen results in altering the mucus barrier in the colon. The only outer mucus layer is present, which is penetrable for microorganisms, which can contribute to colitis development.

Whereas mucus forms the first line of defense coating epithelial cells and contains the same biological components along the whole GI tract, the properties of mucus vary with regional differences. The thickness of the mucus layer differs between the small and large intestine. The major function of the small intestine is digestion of the food and uptake of the nutrients, moreover, the exposure to the microbiota is much lower than in the colon. Therefore, the small intestine expresses a loose, unattached, discontinuous mucus layer that, under experimental conditions, can be easily removed. The detachment of the mucus blanket at a steady state, an essential step for maintaining small intestine homeostasis, is regulated by metalloprotease meprin β that requires the presence of microbiota for its activation [33]. This mucus is also porous and penetrable for different components, including bacteria (Figure 1) [15,34,35,36]. On the other hand, in the large intestine, the thickness of the mucus cover is determined by the number and composition of inhabiting microorganisms. It is organized as two layers: the inner, firm mucus layer, and the outer, loose one. Both layers have almost similar peptide compositions, but there are significant physical differences between them. The inner mucus layer, that remains anchored to the epithelial cells, is highly organized into the flat, lamellar structure, and it does not allow bacteria to penetrate, therefore, at the steady state, it is free from microorganisms. The relative demarcation line divides the inner layer from the outer layer. The outer layer is formed from the inner layer by proteolytic processing of polymerized MUC2, by host bacteria. It thus contains the same components, but is loose, unattached to the epithelial cells, and is inhabited by a large number of intestinal microorganisms (Figure 2) [34,35,37,38]. 

In addition, the transformation from the inner to outer layer is regulated, at least partially, by CLCa1, a metalloprotease with a high abundance in colonic mucus. Nevertheless, studies on a mouse model with *Clca1* deleted gene revealed that, so far, an unidentified cysteine protease can compensate for the lack of the *Clca1* gene. Further elucidation of this mechanism is required [39]. 

### 3.2. Role of Goblet Cells

As it was previously indicated, the major source of mucus is goblet cells. They are present throughout the entire length of the intestine, although they occur in larger quantities in the crypts than on the villi in the small intestine, and in the upper crypts in the colon. Like with other epithelial cells, their differentiation starts within the crypts and is promoted by several transcription factors, including ATOH1 and SPDEF, and also by Notch and canonical Wnt signaling pathways, whereas is negatively regulated by HES1 [28,40,41,42,43].

The goblet cells, migrating from the bottom of the crypts upward the villus, fill their secretory granules, located below the apical membrane, with the main component, MUC2, while the nucleus and other cellular organelles, concentrated in narrow stem-like subcellular regions, are located at the base of the cells [44]. They also synthesize and secrete bioactive molecules, such as secretory and membrane-bound mucins, trefoil factor family peptide 3 (Tff3), resistin-like molecule β (RELMβ), and Fc-globulin binding protein (FCGBP), which all are components of mucus. Tff3 is a tissue-protective factor that promotes epithelial restoration and mucosal repair by apoptosis inhibition, cell migration, and angiogenesis assistance [14,45]. Other proteins, largely originating from the epithelial cells, shed to the lumen, are trapped and present within the mucus biofilm, including: calcium-activated chloride channel 1 (ClCa1), zymogen granule membrane protein 16 (ZG16), anterior gradient 2 (AGR2), and antibodies, especially IgA.

Recent studies focused on the analysis of transcriptomic and proteomic profiles, revealed functional heterogeneity among goblet cell population, defining new functional subpopulations: canonical goblet cells expressing known characteristics for goblet cells genes (e.g., *Clca1*, *Fcgbp*) and non-canonical goblet cells expressing genes connected with enterocytes (e.g., *Dmbt1*, *Gsdmc4*) [32]. 

A new population, named sentinel goblet cells (senGCs), localized at the entrance of the colonic crypt, expressing higher levels of *Il18* and *Nlrp6*, a feature characteristic for non-canonical population, was defined. Studies performed on intestinal explants clearly presented that senGCs, featuring non-specific endocytosis, were able to respond to TLR2/1, TLR4, and TLR5 ligands, leading to NLRP6 inflammasome formation and production of reactive oxygen species, which in turn triggered the release of Ca^2+^ that passed through gap junctions. This resulted in a double-type response: the senGCs secreted their granules and simultaneously sent signals to the neighboring cells. Interesting was that degranulation and MUC2 secretion was sequential. Occurring at first in senGCs, the intracellular signal was then sent through the gap junctions to the closest goblet cells inducing their granules secretion. Consequently, stimulation of goblet cells is progressed around the crypt. Massive MUC2 secretion, initiated by senGCs, was responsible for the physical removal of bacteria from the crypt opening, protecting the lower crypt and ISCs from bacterial invasion. This mechanism was accompanied by the expulsion of activated senGCs to the lumen of the intestine along with remaining endocytosed microbial products [32,46]. 

Mucus secreted by epithelial cells forms a layer separating microbiota as well as potential pathogens, and protects epithelial cells against colonization or even breaching this thin physical barrier. In the colon, it is achieved by the presence of the inner mucus layer that creates the outer mucus layer, which is more accessible for microbiota, while the small intestine is protected by a mucus layer that is penetrable for microorganisms but saturated with AMPs. Any deficiency in mucus formation may lead to colitis. 

In the colon, goblet cells localized between crypts, defined as intercrypt goblet cells (icGCs), are the most differentiated cells, and present expression profiles distinct from the crypt-resident goblet cells. Mucus secreted by icGCs fills the area between colonic crypts and is distinguishable from crypt mucus plume. It is not penetrable for bacteria-size beads but it is permeable for smaller size molecules. This aspect is important in the absorption of ions and other low molecular weight compounds, while denser, impermeable mucus within crypts is responsible for the protection of stem cells area. Both types of mucus form the colon inner mucus layer (Figure 2). However, the significance of inGCs in the development of UC, spontaneous (age-dependent), or chemically induced, was proved in the *Spdef*^−/−^ mouse model, when the formation of functional mucus barrier was altered. Differences in the mucus thickness, composition (crypt goblet cells were the major source of mucus), and the host susceptibility to the development of colitis, due to the alteration of inGCs, were found in comparison to the wild type control. These results correlated with data obtained from patients with diagnosed UC and those in remission. Their intestinal count number of inGCs was reduced, due to the increased cell shedding to the lumen. As a consequence, in those patients, the structural defects in the mucus layer, including gaps in the intercrypt mucus were observed, exposing epithelium to microbial intrusion. These results clearly indicate the role of different subpopulations of goblet cells in providing a tight mucus barrier [32]. 

In addition to their secretory functions, goblet cells play a key role as the luminal sensors of the antigens for the immune system. The question posed by scientists was how the epithelial barrier simultaneously forms a physical obstacle that distances microbiota from the epithelial cells and, at the same time, allows immunocompetent cells that are present in *lamina propria* to selectively sample luminal antigens (microbiota and food antigens) to promote the homeostasis. Beside M cells that can sense and deliver luminal antigens to APCs localized in *lamina propria*, the involvement of goblet cells was elucidated. It was shown that goblet cells after granules secretion, form goblet cells-associated antigen passages (GAPs) that can deliver small, soluble antigens to DCs localized in the *lamina propria*. Since GAPs formation was increased after treatment of goblet cells with cholinergic agonists (secretion stimulators), it suggested that GAPs arrangement was strongly connected with goblet cells mucus secretion. It seems that GAPs formation has an educational impact on the gut immune system by delivering innocuous luminal antigens detected during homeostasis. Recent studies presented that GAPs not only deliver luminal antigens to the *lamina propria* APCs, but also are responsible for maintenance of pre-existing regulatory T cells, imprinting of DCs with tolerogenic features, and promoting *lamina propria* macrophages to produce IL-10. As a result, the formation of the immune tolerance to the dietary antigens is supported. Moreover, in the GAPs absence, this tolerance was impaired, consequently, goblet cells can be considered as the gate-keepers for the orally delivered antigens to the gut immune system [20,47]. 

### 3.3. Role of Enterocytes

The most numerous populations among intestinal epithelial cells are enterocytes in the small intestine and colonocytes in the large intestine. Enterocytes are structurally polarized with the apical surface directed to the lumen and the basolateral surface facing the *lamina propria*. From the apical site, enterocytes form microvilli, actin-based protrusions that increase tremendously the absorptive surface of nutrients, and are described as a brush border [20,25]. Absorptive enterocytes present the pIgR that mediate transportation of dimeric IgA and polymeric IgM from the *lamina propria* across the epithelial barrier to mucosal surfaces [48]. Microvilli facilitate the transport of many molecules (nutrients, electrolytes, vitamins, water, bile salts), however, they are not only in constant contact with microbiota and possible enteropathogens, but are also exposed to mechanical stresses associated with peristalsis. Over the microvillar surface, a carbohydrate-rich glycocalyx is formed, which acts as a protective layer between the mucus and the underlying epithelial cells. Recently, it was shown that the glycocalyx of the brush border is formed of the mucins that belong to the transmembrane mucins family [20,49]. These mucins are synthetized by enterocytes and are characterized by a single domain, passing the plasma membrane, and a large glycosylated extracellular mucin domain, with a similar PTS core as gel-forming mucins (e.g., MUC2). All these mucins also have a cytoplasmic tail that interacts with the cell cytoskeleton. The major components of the enterocyte glycocalyx are transmembrane MUC3, MUC12, and MUC17. MUC1 is more abundant in the stomach and only small amounts of this transmembrane mucin are found in the intestine. The glycocalyx layer comprises epithelial membrane glycolipids and glycoproteins that many act as adhesion receptors for microorganisms [20]. Thus, glycocalyx operates as an attachment site for microbiota and limits the potential colonization area for pathogens. Since the mucus layer in the small intestine, predominantly formed by MUC2 secreted by goblet cells, is a non-attached, relatively porous structure penetrable for bacteria, glycocalyx forms the line of defense. Moreover, the glycocalyx helps in the lubrication and hydrophobicity of mucosal surfaces [25,49].

## 4. Intercellular Junctions—A Protective Barrier

The intestinal epithelium forms a protective lining to maintain the barrier integrity and restrict the entry of microorganisms and toxins, and at the same time, allow penetration of essential ions, nutrients, and water. Even if microorganisms penetrate the mucus layer in the small intestine, the inner layer of mucus in the colon or the glycocalyx, they meet a barrier on the level of epithelial cells formed by a structure composed of three junctions, from apical to basal: tight junctions (*zonula occludens*) (TJs), adherens junctions (*zonula adherens*) (AJs) and desmosomes (*macula adherens*). AJs and desmosomes provide adhesive and mechanical features that contribute to barrier function while TJs form not only tight bonds, constituting links between epithelial cells that physically block microbial invasion, but also seal the paracellular space between the cells and tightly restrict the transport of hydrophilic molecules [50,51]. 

**Figure 3:**
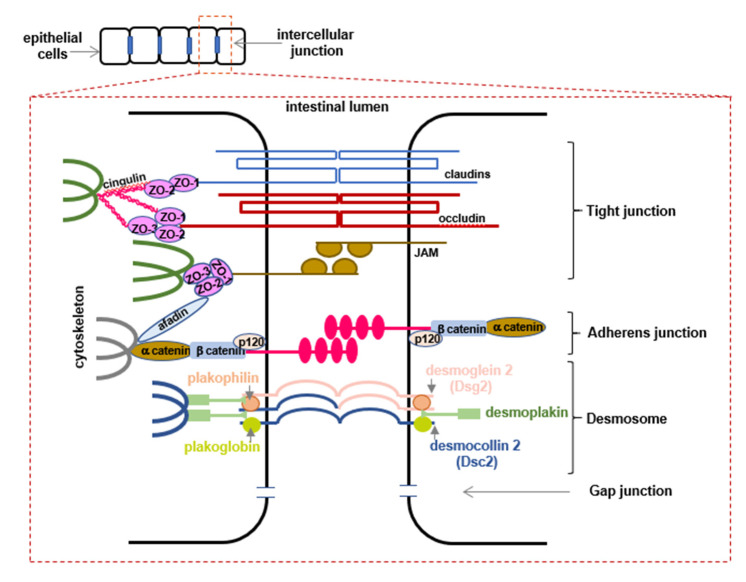
A representative schematic of the intercellular junctions in the intestinal epithelium (according to [5,54,55]). The intestinal epithelium forms a barrier maintained by a structure composed of three junctions: tight junctions (TJs), adherens junctions (AJs), and desmosomes. TJs are generated by membrane proteins, claudins, occludin, and junctional adhesion molecules (JAM) that interact in the paracellular space with proteins on adjacent cells, completely closing the apical intercellular space, blocking microbial penetration, and regulating the selective passage of macromolecules. TJ complex is connected to the actin cytoskeleton through adaptor proteins zonula occludens-1 (ZO-1), ZO-2, ZO-3, as well as other proteins (cingulin, afadine). The lower junctional complexes, AJs, and desmosomes provide adhesive and mechanical properties that contribute to the barrier functions. AJs are created mainly by membrane E-cadherins that form homophilic cell-cell interactions and are intracellularly connected with catenins (α-catenin, β-catenin, p120 catenin) through which they link E-cadherins with a cytoskeleton. Desmosomes are composed of desmoglein 2 and desmocollin 2, forming homo- and heterophilic interactions. They are linked to plakophilins and plakoglobin *via* the cytoplasmatic domain. These in turn are connected to the intermediate filaments by desmoplakin.

AJs are formed between cells by proteins such as catenins, cadherins, and integrins that are involved in cell surface adhesion, providing mechanical strength between cells to facilitate cell-cell adhesion and polarization. Furthermore, they are responsible for binding with the cytoskeleton and thus participate in cellular signaling pathways. One of the catenins, β-catenin, which is involved in cellular adhesion and cell differentiation, also induces Wnt signaling pathway, which is important in gene expression regulation. Cadherins are present on the membranes of adjacent cells binding each other. E-cadherin, the major constituent of AJs, forms homophilic cell-cell interactions and binds intracellularly to catenins (α-catenin, β-catenin, p120-catenin), which links E-cadherin to the cytoskeleton (Figure 3) [52,53].

E-cadherin expression is relatively constant in the small and large intestines, and it contributes to proper cells differentiation and migration from the crypts. Impaired expression of E-cadherin in the small intestine and colon is connected to disturbed gut homeostasis and barrier functions. Overexpression of E-cadherin in the intestine results in retarding cells migration from the crypt to the villus, suppressing their proliferation and induction of apoptosis within the crypt, also delaying the enterocytes differentiation. It has to be said that complete E-cadherin knockout (KO) in mice is embryonically lethal [56], therefore, studies conducted on a mouse model with targeted loss of E-cadherin, somehow elucidated the role of E-cadherin in the GI tract. Loss of E-cadherin causes, among others, accelerated cells migration and aberrant differentiation, as well as villus blunting [57,58,59]. Moreover, the lack of E-cadherin disrupted the paracellular transport pathway, since intestinal epithelium displayed loss of claudin-1 and increased claudin-4 expression, proteins crucial for proper TJs arrangement. Deletion of E-cadherin in adult mice resulted in bloody diarrhea, cell shedding, and deficient cell maturation [5,57,60]. It is clear that E-cadherin is required for maintaining the architecture and function of epithelium since the lack of this protein affects Paneth cells and goblet cells maturation [60]. Moreover, it resulted in reduced AMPs production that led to deficiencies in clearing enteropathogens and predisposed to the development of IBD [61]. 

Desmosomes, similarly to AJs, provide mechanical strength for the cell junctions by comprising transmembrane proteins termed desmosomal cadherins, divided into the desmoglein and desmocollin types (gene names *DSGs/DSCs*; protein names: Dsgs/Dscs, respectively). They are abundant also in tissues of high mechanical stress such as the heart and skin. However, in the intestinal epithelium, only Dsg2 and Dsc2 are present [62]. Both types of desmosomal cadherins are required for cellular adhesion, forming homo- and heterophilic interactions. Dsg2/Dsc2 complex interacts with armadillo proteins plakoglobin and plakophilin, *via* the cytoplasmatic domain. These, in turn, are connected to the intermediate filaments by desmoplakin (Figure 3) [5,63,64]. In vitro studies presented the importance of Dsg2, since *DSG2* knockdown resulted in compensation with Dsc2, but deletion of *DSC2* did not cause changes in Dsg2 protein level [65]. Gross et al. confirmed in an animal model that the desmosomal cadherin Dsg2 is required for the integrity of the GI tract epithelial barrier in vivo. Furthermore, these results have relevance in human diagnostics, since dysregulation of desmosomes was observed in patients with diagnosed CD [63].

Apically located TJ complex is generated by transmembrane proteins and plays a crucial role in maintaining gut homeostasis, as well as controlling the permeability of the paracellular transport pathway. TJs form a continuous network of proteins between membranes of neighboring cells completely closing the apical intercellular space. Additionally, some components of TJs constitute a boundary inside the membrane by itself, restricting the migration of transmembrane proteins and lipids from the apical to the basolateral side and participating in enterocytes polarization [66,67]. The involvement of TJs in the modulation of gene expression, required for cell proliferation and differentiation, was proved to be critical [66,68,69]. The arrangement of the TJ complex is based on the transmembrane proteins that mainly belong to three groups: the claudins family, the Marvel domain-containing proteins (occludin, tricellulin [known as MarvelD2 and MarvelD3 proteins]), immunoglobulin superfamily members (Junctional Adhesion Molecules [JAMs], Coxsackie and Adenovirus Receptor proteins [CAR]). These transmembrane structures are bridged to the actin cytoskeleton and other signaling proteins through a cytoplasmic TJ plaque. TJs plaque is formed by peripheral membrane adaptor proteins, zonula occludens-1 (ZO-1), ZO-2, ZO-3, as well as the cingulin, cingulin-like proteins, and the afadine (Figure 3). TJs establish the backbone of the first line of the host defense by sealing the portal of entry for the pathogens. Various exogenous and endogenous factors regulate the integrity of TJs and, as a consequence, the permeability of the intestine wall. It depends on the involvement of kinase pathways including, protein kinase C (PKC), A (PKA), G (PKG), and MAPK (ERK, p38, JNK) signaling; the calcium/calmodulin-dependent kinase 2 (CaMKK2)-AMP-activated protein kinase (AMPK), as well as Rho and NF-κB pathways [50,67,70]. The regulation and permeability of TJs also depend on the involvement of proinflammatory cytokines, and participation of TNF-α, IL-1β, IL-13, and IFN-γ in the increased gut permeability was highlighted [57,70,71,72]. The structural proteins of TJs play important role in maintaining barrier integrity, although not all proteins functions, so far, were elucidated. Occludin is highly expressed at cell-cell contact and provides structural integrity through the interaction with ZO-1, and phosphorylation of occludin regulates TJs stability as well as permeability [73]. Occludin KO mice expressed morphologically intact TJs structure but exhibited elevated inflammation and a defective gut barrier [74]. Claudins, the family that comprises 23 isoforms, are responsible for the regulation of paracellular space. The extracellular loops of claudin participate in hetero- or homophilic interactions with adjacent cells, which generate barriers or pores for the passage of selective molecules in the paracellular pathway [75,76]. Changes in claudin profiles within TJs, affect the intestinal barrier integrity, depending on the claudin isoform. Therefore, a deficit of claudin-1 in mice leads to the abnormal TJs formation, which induces cancer development and metastasis. Higher expression of claudin-2, a protein required for arrangement of paracellular water channels, together with downregulation of sealing claudin-5 and -8, results in the altered TJs and barrier dysfunction in CD patients and contributes to the inflammation [77,78]. ZO-1, ZO-2, ZO-3 connect claudin and occludin to the actin cytoskeleton, and these proteins interplay maintains TJs function and arrangement. 

TJs in the intestine seal epithelial cells, forming an effective blockade that protects against pathogen intrusion and any alterations in the TJs structure can be detrimental to the host. Some of the examples were already discussed and certain pathological conditions are strongly correlated with a defective intestinal TJs structure such as IBD, obesity (the impairment of intestinal barrier function, alteration in microbiota), nonalcoholic steatohepatitis, and nonalcoholic fatty liver disease (abnormal morphologies of crypts and villi in duodenal mucosa, alteration in microbiota) [70]. However, it cannot be forgotten that this impermeable intestinal barrier creates the target for pathogenic microorganisms, and they developed strategies to disorganize TJs and translocate through the mucosa to invade the host. Paradis et al., thoroughly reviewed the strategies of enteropathogenic bacteria, fungi, and viruses, aiming for the specific proteins, participating in TJs formation. Pathogens modulation of TJs structure can occur through defined molecules, present on the surface of the bacterial or fungal cell walls, bound to the viral capsids, or secreted by microorganisms. In many cases, pathogens employ virulence factors for the disengagement of TJs proteins from the junctional complex. Furthermore, pathogens can induce signaling pathways responsible for TJs and cytoskeleton down- or up-regulation of gene expression, causing disorder of those arrangements. Breaching this barrier by pathogens stimulates immunocompetent cells localized in the *lamina propria*, inducing proinflammatory response as well as oxidative stresses of IECs, leading to the enhancement of TJs dysregulation and this can contribute to the development of IBD [67].

## 5. Paneth Cells—Stem Cell Curators and Microbiota Controllers

One of the major populations, responsible for maintaining homeostasis of the GI tract are Paneth cells, specialized epithelial lineage that resides at the base of crypts of Lieberkühn, and is characterized by the presence of eosinophilic granules in the cytoplasm. Paneth cells were described in the late 19th century, first in 1872 by Gustav Schwalbe, and later in 1888, characterized more thoroughly by Joseph Paneth [79,80]. 

Paneth cells, found only in the small intestine are derived from ISCs, like other intestinal epithelial cells [11,12]. While other, newly formed epithelial cell populations migrate upwards, newly differentiated Paneth cells move downwards further into the crypt, where they mature and start to play their crucial functions in preserving homeostasis of the gut. 

Paneth cells are considered the major regulators of microbial density in the small intestine as well as protectors of stem cells. There are 5–12 Paneth cells per one intestinal crypt with a span life of 3–6 weeks [11,13,81]. These pyramidal in shape cells, with basally located nuclei, are filled with apically localized numerous eosinophilic granules that, upon exposure to Gram-negative or Gram-positive bacteria or their products (lipopolysaccharide, lipoteichoic acids, lipid A, muramyl peptide), release AMPs and enzymes, which are important host-defense factors in the communication between microbiome and the host. It has been proved that bacteria orchestrate secretion by Paneth cells, several proteins and peptides and that include: (i) lysozyme, enzyme active predominantly against Gram-positive bacteria, conducting disruption of glycosidic bonds in peptidoglycan, resulting in bacterial lysis, (ii) secretory phospholipase A2 (sPLA2), showing bactericidal activity against *Salmonella enterica* serotype Typhimurium (*Salmonella* Typhimurium) and *Listeria monocytogenes*, (iii) regenerating islet-derived protein 3 (RegIII) family, that includes RegIIIγ and RegIIIβ, active against Gram-positive bacteria by binding peptidoglycan, component of the cell wall, and critical in the spatial separation of the intestinal bacteria from epithelium in the small intestine, (iv) CRP-ductin responsible for agglutination of Gram-positive and Gram-negative bacteria, (v) enteric α defensins (cryptdins), (vi) cryptdin related sequence (CRS), peptides binding and reducing immunomodulatory activity of LPS, (vii) cathelicidin family (in human the only representative is LL-37) that work similarly to defensins, forming channels in the cytoplasmic membranes, and many other factors like IL-1β, CRIP, IgA [14,79,82,83,84,85,86,87,88]. Enteric α defensins are a subfamily of defensins peptide family that presents a broad spectrum of peptide antibiotic activity, primarily by disrupting microbial cell membranes. Unlike neutrophils, Paneth cells do not store defensins as processed, mature peptides, but maintain them as pro-peptides requiring processing after secretion. In mice, maturation of pro-defensins depends on metalloproteinase (MMP) matrilysin (MMP-7) processing, and mice deficient in this enzyme lack functional cryptdins and are susceptible to oral challenge with *Salmonella* spp. Moreover, the animals had a significantly greater percentage of *Firmicutes* and fewer *Bacteroidetes* in the gut microbiome, compared to wild-type control mice. In humans, instead of MMP-7 that is not present in the small intestine, the involvement of trypsin, as a processor of pro-peptides, was indicated. Moreover, Ghosh et al., showed that trypsin, as a zymogen, was co-localized with human α-defensin5 (HD-5) in Paneth cells granules. These data suggest that trypsin activation occurs either during or after the secretion of granules [79,89,90,91]. Nevertheless, HD-5, the most abundant antimicrobial peptide produced by humans, is shown to have direct bactericidal activity towards distinct members of the human gut microbiota, and thereby it can alter the human microbiome in vivo. More interestingly, HD-5 exposed in vitro to the natural human duodenal fluid underwent proteolytic degradation, resulting in the formation of several active defending fragments, capable of affecting the growth of both, commensal and pathogenic organisms. *In vivo* studies confirmed that HD-5 fragments were able to shift microbiota composition without altering its diversity [92].

Since the epithelial cells are replaced every 2–5 days, the antimicrobial protection of the crypts, the source of the newly formed epithelium, is cardinal, as the damage of ISCs or bacterial outgrowth of the crypts could lead to serious consequences for the host. These granular components of Paneth cells are assembled and packed into dense granules within the endoplasmic reticulum (ER) and Golgi apparatus. It is possible that some granule components are formed elsewhere and are then collected and put together in a granule. IgA are such components that are synthesized by plasma cells in *lamina propria*, accumulated, and then loaded to the Paneth cells granules [79]. Paneth cells activity and health are crucial for microbiome modulation and mediation of the inflammatory response. This secretory cells population is characterized by extensive ER and Golgi network. Endoplasmic reticulum membrane complex subunit 3 (Emc3), encoded in mice by *Tmem111* gene, is involved in proteins folding required for their maturation. Deletion of *Emc3* in the intestinal epithelium affects the differentiation of secretory lineages, goblet cells as well as Paneth cells, resulting in a reduction in the number of the latter ones. The increased level of apoptosis was observed, through which undifferentiated Paneth cells were cleared in Emc3-deficient mice, and that led to a lowering of the final cargo of AMPs, resulting in the growing susceptibility to dextran-sulfate sodium (DSS)-induced colitis and *Salmonella* Typhimurium infection. These results indicate the role of Emc3 in maintaining secretory lineages in the gastrointestinal tract and thereby protecting against the development of inflammation [93].

Nevertheless, the Paneth cells secretory responses remain debatable and the mechanisms that regulate the secretion still are not fully understood. Most of the data, concerning granular contents and secretion by Paneth cells, are based on immunochemistry, and enteroids models, which are three-dimensional cultures of small intestinal epithelial cells [91,94]. It was demonstrated that detection of enteric bacteria by Paneth cells occurs through the MyD88-dependent pathway, but TLR4 independent way [95,96,97,98]. Moreover, the presence of microbiota was required for Paneth cells activation. Studies, performed by Vaishnava et al. on mice, in which Paneth cells were specifically ablated by expressing a diphtheria toxin fragment under the control of cryptdin-2 promoter (CR2-*tox176* mice), provided information concerning the involvement of Paneth cells in limiting mucosal penetration by microbiota and pathogenic bacteria. Although in both wild type and Paneth cell-deficient mice the luminal bacterial load was comparable, the number of bacteria recovered from MLNs was higher in CR2-*tox176* mice. Similar results were obtained when germ-free (GF) CR2-*tox176* mice and GF wild type mice were orally introduced with *Bacteroides thetaiotaomicron*, and in a parallel experiment, when mice were challenged with *Salmonella* Typhimurium. These results emphasized the importance of Paneth cells as a mandatory population for limiting pathogens translocation from gut lumen and their dissemination to the tissues [95]. 

Studies, performed on the three dimensional enteroid models, shed new light on Paneth cells activation and their functions. Since enteroid is a closed structure, with Paneth cells apical sites opened to the lumen of the enteroid, the microinjections with bacterial stimuli (LPS, or *Salmonella* Typhimurium *phoP*), were used to evaluate their activation. This experimental model clarified the ability of Paneth cells for a rapid response with AMPs secretion (within 2–14 s), upon only apical stimulation. Moreover, the potential of quick refilling of granule store (21 h post secretion), with newly generated granules and ability to respond to secondary stimuli with comparable strength, was demonstrated. 

However, basolateral site stimulation is also responsible for Paneth cells induction, leading to the AMPs secretion, but in this pathway, stimulation with, among others, muscarinic nerve stimuli, cytokines (IL-13 and IL-22) are involved [91,99,100]. 

Various bacterial components, such as LPS, non-methylated CpG oligodeoxynucleotides, and muramyl dipeptides, induce Paneth cells secretion that is followed by regulation of the number of bacteria in the intestine. Although the involvement of microbiota and pathogens in Paneth cells stimulation and subsequent granule release is unquestionable, the other stimuli, controlling their ability to AMPs secretion, should be considered. IFN-γ, a strong modulator of the immune response, was a candidate for Paneth cells activation. However, in vitro studies revealed that exposure of enteroids to this cytokine caused Paneth cells death through caspase 3/7 pathways shortly post exposure. These results are in accordance with other studies, both in vivo and in vitro. They indicate that IFN-γ caused a rapid and complete Paneth cells degranulation, followed by their luminal extrusion and apoptosis. Such a strong degranulation and loss of Paneth cells leads to dysregulation of microbiota control and results in dysbiosis. Moreover, this feature is shared with goblet cells, since depletion of both cell populations (Paneth cells and goblet cells), during infection is induced by IFN-γ. Some mechanisms, regulating granule releasing were defined, however, the exact stimuli that induce Paneth cells to secrete AMPs have to be elucidated [14,91,101,102,103,104]. One of the proposed hypotheses suggests that at first there is an impact of AMPs on bacteria that are apposed to the mucosal surface since bacteria must be mucosa-associated before the uptake by DCs for MLNs translocation or before the direct invasion of epithelium. This is consistent with the fact that AMPs are retained by the mucus layer that overlies the epithelium. Moreover, secreted lysozyme and cryptdins can be recovered as intact, functional form from the lumen of the small and large intestines as well as from feces, suggesting that these factors affect the microbiota composition of both the small intestine and colon, and contribute to the intestinal homeostasis. By specific controlling of the bacteria-mucosal surface interactions, Paneth cells contribute to maintaining the proper composition of luminal microbiota that is essential for the host metabolic health [91,95,105,106]. 

As discussed, Paneth cells are the major source of AMPs participating in shaping host microbiota. On the other hand, they can be the origin of intestinal inflammation in the host. This point of view has some relevance since the recent studies link the reduced number of Paneth cells or their abnormal function with microbial dysbiosis in the ileum, detected in patients diagnosed with CD. Paneth cells are identified as the site of susceptibility gene defects that impair the secretion of Paneth cell granules. The mutations of CD susceptibility genes *Atg16/1* and *Xbp1* are linked with defective granule exocytosis from Paneth cells with diminished levels of defensins, due to the abnormal autophagy and ER stress that affects the maintenance of secretory cells [13,91,107,108]. NOD2, a cytoplasmic PRR belonging to NLRs, present in Paneth cells cytoplasm, is critical for regulation of the bacterial microbiome in the ileum through secretion of AMPs. Mutations in the *NOD2* gene affect the C-terminal leucine-rich repeat receptor domain that recognizes and binds muramyl dipeptide leading to deficiency in sensing bacteria in the ileum. As a result, the abnormal microbe colonization of the small intestine occurs, which may provoke chronic inflammation, since Paneth cells with *NOD2* mutation show reduced secretion of α-defensins. *Tcf4* gene mutation of Wnt signaling pathway transcription factor that orchestrates Paneth cells differentiation, is directly linked with impaired cell maturation and diminished α-defensins production, and its corresponding antimicrobial function [13,14,91,108,109]. Microbial dysbiosis is one of the factors of obesity pathogenesis. A strong connection between the pathology of such disease and Paneth cells was found since HD-5 and lysozyme expression was significantly decreased in Paneth cells in obese patients comparing to healthy subjects of normal weight [110]. Furthermore, Paneth cells dysfunction is associated with enteropathy in graft-versus-host disease (GVHD). In GVHD, the intestinal tract is frequently affected, as a result, Paneth cells loss is a crucial point in shifting to microbiota dysbiosis. The GVHD mouse model developed dysbiosis due to depletion of Paneth cells that resulted in the loss of secreted α-defensins, but can be partially reversed after oral administration of α-defensins and that improves GVHD survival [111,112]. Paneth cells develop in the middle of human gestation, however, they do not rich their density and immunocompetence until closer to the term of gestation. Preterm neonates lack fully differentiated Paneth cells and they show great susceptibility for the development of intestinal pathology, such as necrotizing enterocolitis (NEC) [13,79]. 

Although Paneth cells are localized in the base of the crypts and are confined to the small intestine, in distinct intestinal disorders they can appear in various areas, in which they are not normally found. Paneth cell metaplasia is seen throughout the gastrointestinal tract but can be also found in extra-gastrointestinal sites, like the lungs, tracheobronchial system, urogenital tract, pancreaticobiliary tract, and rarely in the nasopharyngeal system [13,113]. So far, the mechanism of Paneth cells metaplasia is not fully comprehended. The Wnt/β catenin canonical signaling pathway is required for full differentiation of Paneth cells. There is some evidence that this signaling is involved in metaplasia development [41]. Moreover, additional components required for Paneth cells differentiation that differ between tissues can be involved in this mechanism, where inflammation that can induce metaplasia/hyperplasia, seems to be one of them. Indeed, chronic inflammation usually precedes the formation of intestinal metaplasia, like in the case of Barrett’s esophagus, or in the stomach, following *Helicobacter pylori* infection. Paneth cells metaplasia occurs in the chronic inflammatory state within the colon (UC, colonic CD, diverticulitis), and it was noticed that metaplastic Paneth cells express their regular AMPs. It was suggested that the occurrence of metaplastic Paneth cells at mucosal sites, either intestinal or extra-intestinal, probably represents a protective, antibacterial and inflammatory response evoked by an altered microbial activity [2,13,113,114,115]. 

Studies on the relationship between intestinal cells and ISCs are based on the application of organoid technique and animal model. Thanks to these studies it was possible, at least partially, to elucidate the mechanisms responsible for maintaining intestinal homeostasis. The base of the crypt is an intrinsic pattern of crypt base columnar cells expressing *Wnt* target gene, leucine-rich repeat containing G protein-coupled receptor 5 (Lgr5^+^), in close contact with Paneth cells that are the source of secreted proteins, such as Wnt3, epidermal growth factor (EGF), and Notch ligand Delta-like (Dll) 4 and Dll1 that are crucial for stem-cell support [11,14,87,116]. Due to their proximity to Lgr5^+^, Paneth cells affect the function of ISCs activating the canonical Wnt/β-catenin signaling pathway by delivering Wnt3 that is bound by Frizzled receptors to improve the function of Lgr5 stem cells [101,116,117]. Studies conducted on the organoid model indicate the role of EGF in ISCs proliferation. Paneth cells and mesenchymal cells are the major source of EGF with EGFR expressed on ISCs. Lack of EGF in cytokine environment or blocking EGFR signaling pathway by EGFR Gefitinib inhibitor subdues ISCs proliferation. However, the proliferation process of ISCs is more than that regulated by Lrig1, expressed thereby cell population, which serves as a cell surface negative regulator of EGFR/ErbB pathway [12,116,117,118]. 

Notch signaling is dependent on cell-to-cell contact of membrane bound Notch ligands on one cell, and Notch receptors (Notch 1 and Notch 2) in neighboring ISCs. Dll4 and Dll1, produced by Paneth cells, maintain ISCs proliferative ability. Abrogation of both Notch ligands leads to the complete conversion of proliferative progenitors into post-mitotic goblet cells [79,119,120]

The relationship between Paneth cells and ISCs was much harder to prove in vivo. Nevertheless, the conditional deletion of Sox9, a protein required for Paneth cells differentiation dependent on the Wnt signaling pathway, in the animal model, allowed to confirm the role of Paneth cells as a source of supplying signals for stem cells as well as proved that the cell-cell contact facilitated cell homeostasis. Subsequent reduction of Paneth cells in the crypts resulted in a withdrawal of ISCs, implying that their maintenance depends to a large extent on the presence of Paneth cells [94,121,122,123].

Paneth cells are able to participate in the regeneration of the epithelial layer. Mature, terminally differentiated Paneth cells are featured by the presence of CD24, lysozyme, and MMP7. Upon epithelial injury, followed by an inflammation, when the quick rebuilding of barrier is required, Paneth cells are able to acquire stem cells characteristics through de-differentiation *via* Notch and SCF/c kit signaling, contributing to the epithelium reconstitution [12,116,124,125,126]. The plasticity of Paneth cells was proved in studies performed on irradiated mice. Intestinal Lyz^+^ cells collected from irradiated mice, seeded and maintained under in vitro conditions were able to enter the proliferative state followed by differentiation into villus epithelial cells. Moreover, these Paneth cells-derived epithelial cells did not express Paneth cells markers, such as MMP7 and Lyz^+^. Inflammation of the intestine wall often is accompanied by the loss of Paneth cells [125,127,128]. Studies conducted by Yu et al. suggest a high possibility that the diminished number of Paneth cells, noticed in various diseases, may be the consequence of Paneth cells de-differentiation. It is worth mentioning that, in addition to Paneth cells, other differentiated intestinal epithelial cells can obtain stem cell features upon specific conditions and participate in epithelial damage repair [125]. 

The Paneth cells participation in aging cannot be excluded. The aging in the GI tract is represented by decreasing balance between stem cell reserve and differentiation. Animal models, as well as organoid technique, clearly present that during physiological aging the reduction of crypt number along with the increase in the crypt length and width is observed. Additionally, the aging of ISCs is driven by mTORC1 and canonical Wnt signaling in ISCs is decreased. However, the expression of genes encoding Wnt3 or EGF in old Paneth cells is not significantly altered, so their influence on the ISCs is not achieved through the Wnt signaling pathway [129,130,131]. However, at the same time, a significant upregulation of Notum, the extracellular Wnt inhibitor responsible for negative-feedback loop formation, occurred in evaluated Paneth cells in aging mice. It shows that by producing Notum, aging Paneth cells can affect the regenerative capacity of stem cells by silencing the Wnt signaling pathway [132,133]. 

Paneth cells are a group of terminally differentiated epithelial cells, localized at the base of the crypts, and contain apical secretory granules filled with AMPs that are mandatory for controlling intestinal microbiota. Moreover, their localization and proximity of ISCs regulate the function of ISCs throughout paracrine-specific proteins secretion. In the pathological state, Paneth cells express plasticity and undergo de-differentiation into stem cells supplying the pool of ISCs. By shaping the host microbiota and controlling ISCs, they prevent the development of intestinal inflammation and maintain gut homeostasis.

## 6. Cross-Talk between Microbiota and Epithelial Cells, a Step Required for the Maintenance of the Intestinal Barrier

The GI tract is inhabited by an enormous number of microorganisms, termed gut microbiota, and they form a mutualistic relationship with the host. First, the presence of microbiota in the GI tract is beneficial, but at the same time those microorganisms create a permanent threat to the host, therefore preventing their translocation into the underlying tissue is of paramount significance. 

Thus, the integrity of the GI tract barrier is vital for maintaining the health of the host. The critical role in barrier arrangement is played by epithelial cells, simultaneously participating in the segregation of intestine microbiota and possible pathogens by forming physical and chemical obstacles. At the same time, epithelial cells perform mediation by sensing the microorganisms and, as a result, secreting mediators, including cytokines and chemokines, that will stimulate or inhibit the immunocompetent cells located in *lamina propria*, leading to inflammation or immune tolerance induction. However, to form physical and chemical barriers, epithelial cells require continuous cross-talk with the GI tract microbiota. Any impairment in barrier functions can be associated with the uncontrollable immune reaction in the intestine and/or promotion of the unrestricted growth of microbiota, which leads to various diseases, including inflammatory and metabolic disorders, such as IBD, obesity, or even cancer [24,32,36,134].

### 6.1. Sensing and Recognition of Microorganisms

As it was discussed previously, a physical barrier is formed by mucus, secreted by goblet cells, with major participant MUC2, and by intercellular junctions. Mucus that covers the GI tract is a part of the innate mucosal barrier and prevents inflammation by limiting the antigen exposure as well as bacteria to the immune cells underlying the epithelial layer. It creates a diffusion barrier, through which small molecules such as ions, nutrients, gases, water can penetrate, and reach the epithelial cells. Moreover, it protects against mechanical, chemical, and biological attacks, also it works as a lubricator to facilitate the passage of cellular debris, bacteria, and fecal material, flushing them away through the intestinal channel [15,24,31,135,136,137]. 

The physical barrier can be increased by defense mechanisms induced as a result of microbial sensing and recognition by IECs that carry out PRRs, especially TLRs and NLRs, which recognize MAMPs or DAMPs released from the host cells. TLRs are expressed in most IECs lineages, including stem cells, enterocytes, goblet cells, enteroendocrine cells, Paneth cells, M cells, and they play an important role in the detection of microorganisms under pathologic as well as homeostatic conditions [3]. The distribution of TLRs varies and changes along the intestine, with small intestinal IECs expressing lower levels than colonocytes, which correlates with a much lower number of microorganisms than in the large intestine [96]. To maintain the GI tract homeostasis, IECs had to develop a tight TLR-regulating system to avoid disproportionate reaction towards gut microbiota. These cells can control and modulate TLR-signaling at different levels, such as by restricting access to the ligands or by inactivating downstream cascade.

Enterocytes are structurally polarized with the apical surface facing the intestinal lumen, and the basolateral site connecting with the *lamina propria*. Enterocytes express TLR2, TLR3, TLR4, TLR5, and TLR9, with the majority of TLRs present at the basolateral membrane, while TLR2, TLR3, and TLR9 are also expressed at the apical surface [138,139,140,141]. As a consequence, TLRs activation can lead to different cellular responses, depending on the localization of the sensing receptors. Basolateral TLR stimulation results in a signaling cascade that leads to the NF-κB nuclear translocation, subsequently, expression and secretion of cytokines and chemokines, including TNF-α, IL-6, IL-12, IL-18, CXCL8, CCL20, which activate immunocompetent cells localized in *lamina propria*. Such activation and induction of inflammatory response are the results of breaching the epithelial barrier by pathogenic microorganisms [14]. This mechanism was explained in the elegant experiments presented by Gewirtz et al., indicating the requirement of crossing the epithelial barrier by flagellated bacteria to be recognized by TLR5. TLR5 recognizes flagellin, the structural component of bacterial flagella, and is critical for the detection of invasive flagellated bacteria at the mucosal surfaces. Only flagellated bacteria, that were able to cross the mucosal membrane, induced inflammation, while flagellin of luminal, commensal *Escherichia coli* strains did not evoke a comparable effect [142]. TLR9 is presented on the apical and basolateral surface of IECs and recognizes unmethylated CpG sequences expressed at high levels in prokaryotic DNA found within the commensal microbiome as well as within prokaryotic pathogens, invading the GI tract. Detection of luminal unmethylated CpG of microbiota *via* apically located TLR9 results in stabilization of IκB (inhibitor of NF-κB), and making IECs hyporesponsive to apical interaction to TLR9 ligands. However, once pathogenic bacteria break through the epithelial barrier and unmethylated CpG sequences are detected *via* basolateral TLR9, NF-κB activation occurs, subsequently leading to the proinflammatory cytokines and chemokines production [143]. The maintaining of the physical barrier relies on mucus production and secretion. Moreover, the density of mucus largely depends on the presence of bacteria. In MUC2^−/−^ mice there is no inner mucus layer physically segregating microbiota and colonocytes, and as a result of their direct contact with microorganisms, the inflammatory response is provoked and spontaneous colitis is induced [36,37,144].

The correlation between the sensing of microorganisms and mucus secretion was discussed earlier, in the context of senGCs that respond through TLR1/2, and TLR4 and TLR5 ligands leading to the increased MUC2 secretion, and enforcement of mucus barrier [46]. Probably disruption of this mechanism is the explanation of the altered microbiota composition and increased susceptibility to the development of spontaneous colitis observed in TLR5^−/−^ mice. However, mice without TLR5 express mosaic phenotype, with a subset of mice that shows increased susceptibility to the spontaneous colitis development and lack of the normal, double structure of colon mucus layer. Only a disorganized mucus layer that is penetrable for microorganisms is present. Non-colitic TLR5^−/−^ mice had normal, though slightly thinner mucus layer. Studies of another group indicated that deficiency of TLR5 altered the composition of the intestinal microbiota in comparison to the wild type mice, and low-grade inflammation and susceptibility to colitis were observed [145,146]. Furthermore, TLR1 is involved in mucus synthesis, since a large area of the colon with a patchy and significantly depleted mucus layer is observed in TLR1^−/−^ mice, as a result of a defective production and/or secretion of MUC2 [147]. Additionally, increased intestinal inflammation and overgrowth of *Candida albicans* and *E. coli* in the colitis model were observed in mice with TLR1 deficiency [148]. 

Furthermore, commensal bacteria, upon interaction with TLRs, are responsible for the enforcement of the epithelial barrier. One of the species is *Akkermansia muciniphila*, the Gram-negative bacteria, inhabiting the human GI tract, producing mucin-degrading enzyme, and utilizing mucins as a source of energy. Within the outer membrane, it presents a protein Amuc_1100, interacting with TLR2, and thereby interplay is able to modulate the gut barrier and the intestinal permeability by increasing mucus thickness and TJs proteins, such as occludin, claudin 3, and cannabinoid receptor 1 [149,150]. These data clearly show that TLRs involvement is important in the development of a healthy mucus layer in the GI tract.

### 6.2. Factors Influencing the Colonization of the GI Tract with Microbiota

Microorganisms are spatially organized along the length of the intestine and distributed in the lumen according to the oxygen levels and nutrients availability. The gradient of microorganisms increases from the proximal to the distal GI tract, and from the epithelial layer towards the lumen [24,28,151]. Moreover, the viscosity of the mucus increases toward the distal regions of the GI tract. In addition, since mucus, at a steady state, is constantly secreted by goblet cells, it creates a continuous flow towards the lumen that, together with AMPs and IgA present in mucus, keeps microorganisms apart from the epithelium. Mucus determines the distribution and organization of the microbiota in the intestine and protects against the bacterial colonization of epithelial cells and crypts. However, for proper arrangement of the intestinal mucus, the presence of microbiota is absolutely required. Studies performed on the GF mice and antibiotic-treated mice highlighted the importance of interaction between goblet cells and microorganisms. The early studies indicated the thinner layer or even local lack of mucus in the colon of the GF animals compared to the conventionally raised (Convr) rats. The mucus in the colon was penetrable to bacteria. Moreover, the replication of epithelial stem cells was disrupted and antibiotic-treated mice were more susceptible to colitis, induced physically or chemically [24,152,153]. Since comparison of the GF rodent model with Convr animals gave some insights to the formation of mucus protective layer, in nature we are facing a natural model that can be, at least initially, compared with GF animals. The colonization of the intestine with individual microbial populations in neonate animals can also serve as a model for analysis of the microbial influence on the development of mucus. Indeed, the increased expression of genes encoding Muc1, 3, 4, membrane-bound mucins was observed even in the absence of microbiota, between 1–6 postnatal days. However, the increased expression of the gene, encoding secreted MUC2, required the presence of microorganisms. Moreover, mice monocolonized with the probiotic bacteria (*Lactobacillus acidophilus* or *E. coli* Nissle 1917), exhibited a similar gene expression profile to the neonate GF mice, indicating that the complex microbial population is required to stimulate the *Muc2* gene [154]. Moreover, bacterial products (LPS, peptidoglycans), can be involved in the restoration process of mucus secretion, since the GF mice treated with those compounds obtained a functional mucus protective layer, comparable to the Convr mice [155]. It can be said that the protective mucus layer in the small and large intestines depends on the presence of microorganisms and can be rebuilt since the impenetrable layer of mucus that served as a barrier against microbiota translocation occurred within 5 weeks in GF mice after colonization with a complex microbial community [156]. 

In the small intestine, where mucus is thinner and penetrable for microorganisms, or there are intended loopholes within the mucus layer, the microbiota is controlled and shaped due to the presence of AMPs, the major source of which are Paneth cells. This chemical barrier formed by the defensin family of proteins, cathelicidins, RegIII family, lysozyme, RELMβ, and IgA has a critical role in the segregation of intestinal bacteria and epithelial cells, suppressing colonization and overgrowth of the microorganisms. Using the enteroid culture technique Schoenborn et al., indicated a diminished number of Paneth cells in the GF mice in comparison to the Convr animals. Moreover, the alterations in RegIIIγ transcript levels in Paneth cells were significantly reduced in the GF small intestine crypts, relative to those in the Convr animals. Meanwhile, the enteric microbiota did not influence the number of ISCs [157]. Therefore, the presence of intestinal microorganisms affects the number of Paneth cells and hence, the integrity of the epithelial barrier, since Paneth cells regulate stem cells homeostasis. In addition, the dependence of defensin cryptdin 2 and RegIIIβ and RegIIIγ production was demonstrated by Vaishnava et al., indicating a reduction of AMPs in Myd88^−/−^ mice [95].

Furthermore, sIgA, the hallmark of mucosal immunity, are secreted in response to luminal antigens delivered to immunocompetent cells located in *lamina propria*. The major entrance gates for such antigens are areas devoid of mucus in the small intestine that are localized above Peyer’s patches and are characterized by the presence of M cells (Figure 1). M cells are not strict APCs in the context of DCs or macrophages, but they are antigen delivering cells that transfer luminal particles and antigens to the *lamina propria* DCs for antigen presentation, leading to the immune response or tolerance induction. Constant contact of M cells with microbiota results in B cells stimulation for IgA production, their transport to the intestine lumen using pIgR, and, as a result, control of microbiota by sIgA through the aggregation of bacteria and prevention of mucosal barrier crossing by size exclusion [3,18,158]. Glycoprotein 2 (GP2), a transcytolytic receptor present on M cells surface, is involved in the antigen uptake. Antigen-specific IgA response is suppressed in mice lacking GP2 [159,160]. It is worth noticing that the expression of pIgR, involved in IgA transcytosis, is MyD88-dependent since in vitro studies revealed upregulation of pIgR and increased transcytosis of IgA upon stimulation of epithelial cells with LPS or heat-killed *E. coli*, indicating TLR-mediated recognition dependence [161,162]. 

Although AMPs secreted by Paneth cells can be identified in the colon or even in feces in their native form, their expression level is not high compared with a small intestine, and it is not clear what compounds participate in the segregation of microorganisms in this area. The inner mucus layer is involved in this mechanism since at the steady state, this barrier is free of microbiota and not penetrable for microorganisms. However, the mechanisms that separate bacteria and colonocytes are not fully elucidated. Early events of colonization often depend on the flagella-mediated motility of bacteria. Ly6/Plaur domain-containing 8 (Lypd8) protein, highly expressed on colonocytes and constitutively secreted to the lumen is a novel molecule, contributing to the segregation of microorganisms in the colon. This molecule participates during the early-phase protection by preventing colonization of the large intestine by flagellated bacteria, such as *Proteus mirabilis* and *E. coli via* flagella binding, thereby suppressing colonic epithelium invasion [163]. The parallel mechanism, engaged thereby molecule, was recently elucidated by Okumura et al. using a *Citrobacter rodentium* model, frequently used for clarification the mechanisms of pathogenesis of human infections with enteropathogenic *E. coli* (EPEC) and enterohaemorrhagic *E. coli* (EHEC). The invasion of the intestinal mucosa with enteropathogenic bacteria routinely depends on the presence of virulence factors, among which, type III secretion system (T3SS) is involved, forming a link between bacterial cytoplasm and a target host cell cytoplasm. Following the maturation of the T3SS translocon, translocated intimin receptor (Tir) is exported by the bacteria and integrated into the host cell plasma membrane. Intimin, a bacterial adhesion molecule involved in an intimate attachment of enteropathogens, interacts with Tir, which plays a central role in actin condensation beneath the adherent bacterium, required for characteristic, pedestal-like structures formation. Those structures are known as the ‘attaching and effacing’ (A/E) lesions on the IECs and promote tighter binding of bacteria to epithelia [164,165]. Lypd8 shed by colonocytes, suppressed the attachment of *C. rodentium* to colonocytes by inhibition of the interaction between intimin and Tir. It is competitively bound to intimin, effectively blocking Tir-intimin interplay, and is required for the generation of A/E lesions. More rapid intestine colonization with *C. rodentium* and more severe colitis in Lypd8^−/−^ mice were observed. Interestingly, human Lypd8 bound to EHEC intimin indicates that the ability of Lypd8 proteins to connect with intimin of A/E bacteria is conserved. These data emphasized the importance of the enterocytes in the protection of the integrity of the intestinal barrier [36,166]. In addition, a lectin-like protein ZG16 that specifically binds peptidoglycan of Gram-positive bacteria and thereby inhibits their penetration into the inner colonic layer is present in the colonic mucus [167].

### 6.3. Mutual Dependency of IECs and Intestinal Microbiota

Mucus in the small and large intestines, engaging different, yet effective protective mechanisms, maintains segregation of microbiota from epithelial cells and thereby prevents inflammation. On the other hand, the presence of gut microbiota in the mucus is required, since microorganisms are the source of many metabolites, beneficial for the host physiology. At the same time, mucus is the source of the energy, and thereby it can shape the gut microbiota supporting the GI tract homeostasis. The composition of the gut microbiota can undergo changes from mucosal to the luminal site, forming different ecosystems. Their complexity can be affected by many factors, such as hygiene, diet (especially “Western diet” low in fiber and high in sugar and fat), oxygen concentration, mucus, microbial adherence, antimicrobial compounds, host stress, and immune response, leading, in many cases, to dysbiosis [24,28,31]. The GI tract microbiota degrades dietary substrates that are not used and absorbed in the small intestines and thus reach the colonic lumen. They are usually plant-derived polysaccharides, for which the host presents a rather limited enzymatic profile of approximately 17 carbohydrate-active enzymes (CAZymes) as opposed to, at least, human microbiota-encoded 89 CAZymes families, suggesting the ability to digest a huge range of carbohydrates [168]. Though gut microbiota feeds on dietary fibers non-digestible for the host and produces compounds that exert positive effects on the intestinal mucosa, the mucus layer is an alternative source of host-derived glycans. Mucus creates the selective niche, which serves as an attachment site for microorganisms, broadly described as “mucus-associated microorganisms”. Additionally, mucin glycans provide nutrients for microorganisms called “mucolytic bacteria”, supporting their growth and colonization as well as offering sources of carbon and energy. These bacteria digest glycan using exoglycosidases that allow removing one sugar residue per time, and when all glycans are removed, the protein core of the mucin is degraded [34,151]. This process leads to MUC2, and, finally, mucus degradation. Not all bacteria are able to remove glycan residues, therefore removed glycans can be used by bacteria and other members of gut microbiota that digest them. Among specific enzymes essential for mucin degradation are sialidases, fucosidases, sulfatases, proteases, belonging to the category of carbohydrate-active enzymes. Such metabolic plasticity is evident and beneficial for microbiota when complex dietary carbohydrates are missed in the diet. This ability of switching from dietary glycans to mucus glycans determines which gut microbiota can survive when supplementation of dietary fibers is reduced. However, such diet deprivation can have serious consequences for the host. 

It was proved in the mouse model that animals fed a fiber-free diet presented decreased thickness of mucus layer that increased their susceptibility towards infection with *C. rodentium* and that effect was similar to the MUC2^−/−^ mouse model, where the colon inner mucus layer was thinner, the proximity of gut microbiota to the epithelial cells was smaller, and as a consequence, the development of spontaneous colitis was observed [31,37,144,169]. Hence, the strong correlation between diet-dependent loss of mucus layer resulting from the lack of MUC2, and protection against infection by enteric bacteria is based on the involvement of gut microbiota in forming a protective, impervious, and functional barrier.

Mucus degrading bacteria present within mucus include *Akkermansia muciniphila*, *Bacteroides thetaiotaomicron*, Bifidobacterium bifidum, *Bacteroides fragilis*, *Ruminococcus gnavus,* and *Ruminococcus torques*. Those species generate short-chain fatty acids (SCFAs) through a fermentation process, using glycans as an energy source [24,28]. SCFAs are carboxylic acids, produced by anaerobic fermentation of dietary fibers in the intestine, of which acetate and propionate are produced by *Bacteroidetes* (Gram-negative bacteria) while butyrate is produced by *Firmicutes* (Gram-positive bacteria) in the human gut [169]. 

Those compounds are then absorbed and used by colonocytes to recover part of the energy spent for MUC2 synthesis. Furthermore, in the GF or antibiotic-treated mice fed a diet supplemented with a mix of SCFAs, the proliferative activity of small intestine IECs was restored. It indicates the role of the GI tract microbiota in maintaining homeostasis of the intestine [170]. However, this activating influence depends on the type of the cells and concentration of SCFAs, since butyrate had an inhibitory effect on ISCs. It seems that this is a kind of safety mechanism, protecting against uncontrollable cell divisions, as long as sodium butyrate is used in colorectal cancer cell lines, suppressed cancer cell line proliferation, and induced apoptotic cell death [171]. There is a close relationship between the maintenance of anaerobic conditions necessary for SCFAs-producing bacteria and the strengthening of the epithelial protective barrier. Butyrate is the main energy source of colonocytes, which consume more than 70% of oxygen due to butyrate oxidation and thereby, support maintaining anaerobic niche. Depletion of anaerobic bacteria, source of SCFAs (due to antibiotic treatment or fiber-low diet) induces colonocytes to undergo anaerobic respiration, which causes a release of oxygen and nitrates to the lumen. This mechanism results in promoting anaerobic environment and inducing overgrowth of facultative anaerobic pathogenic bacteria e.g., *E. coli* and *Salmonella* spp. [172,173,174]. Moreover, it is possible that butyrate can enforce the epithelial barrier by inducing gene expression encoding proteins of TJs, as in vitro studies revealed these effects [175]. Acetate produced by *Bifidobacterium longum* protects IECs against apoptosis induced by the O157 toxin. In addition, acetate induces goblet cells differentiation, mucin secretion, and their sialylation [153]. Dysbiosis, which is frequently observed in patients diagnosed with UC or CD, leads to a diminished number of bacteria producing SCFAs. Deficiency in those metabolites within the intestine results in the weakening of the protective barrier, the functionality of epithelial cells as well as mucus production. As a result, unwanted overgrowth of microorganisms occurs that can lead to an unrequired inflammatory response. 

In addition, other microbe-derived metabolites are involved in maintaining epithelial cells functionality. Lactate is a potent inducer of small intestine Lgr5^+^ cells, causing their hyperproliferation. Lgr5^+^ cells are supported by Paneth cells with various factors (Wnt3, Dll1, Dll4, EGF) required for maintaining proper cell functions. Using the organoid model it was revealed that Paneth cells support Lgr5^+^ cells functions by providing lactate to aid enhanced mitochondrial oxidative phosphorylation, which is required for establishing mature crypt phenotype through signaling [176]. However, even though the importance of this metabolite in maintaining crypt homeostasis was emphasized, it is still controversial whether lactate producing bacteria, e.g., *Lactobacillus* spp., affect epithelial stem cell homeostasis in vivo. Nevertheless, intestinal microbiota and microbiota-derived metabolites are important for maintaining the epithelial barrier integrity and homeostasis. Moreover, this cross-talk between epithelial cells and commensal microorganisms prevents the development of host dysfunctions.

## 7. Conclusions

The GI tract is a place that is constantly exposed to a multitude of stimuli, however, homeostasis of this area, and by that means homeostasis of the host, is maintained. In this review, we focused on the role of specific epithelial cell populations engaged in the formation of the first line of defense against mucosa colonization with pathogens as well as their mutualistic relationship with the GI tract microbiota. It summarizes the data of many investigators, working on all aspects concerning mechanisms involved in preventing the development of inflammation in the GI tract. IECs, due to their localization between the GI tract microbiota and immunocompetent cells found in *lamina propria*, play important role in transferring signals responsible for silencing immune response and inducing tolerance directed towards commensal microorganisms and food antigens. Maintaining the integrity of the barrier, based on the mucus secretion by goblet cells and enterocytes as well as formations of the intercellular junctions between epithelial cells, is crucial for the prevention of inflammatory response due to the spatial segregation of microbiota. However, as a major producer of AMPs, Paneth cells have a strong influence over microbial composition in the small intestine. Moreover, through their localization at the base of the crypts of Lieberkühn and proximity of ISCs, Paneth cells regulate the function and differentiation of crypt stem cells, subsequently affecting the physiology of newly formed epithelial cells. Nevertheless, cross-talk between the IECs and gut microbiota is required to maintain the homeostasis of the intestine. It is comprehensively demonstrated that members of gut microbiota are not the passive bystanders in the GI tract, but instead are active participants in establishing homeostasis in the GI tract. TLR interactions regulate the barrier formation, inducing/silencing immune response, controlling the permeability of the barrier, and stimulating the secretion of AMPs. The formed mucus layer serves not only as a barrier but also as an attachment site and the source of energy for microbiota, creating a microecosystem that influences the physiology of the host through SCFAs secretion by commensal bacteria. The GI tract microbiome is a very complex community that can be altered by many factors, including antibiotic treatment as well as a diet. Recently, the importance of a fiber-rich diet as a vital factor affecting the profile of the gut microbiome and the interplay of microorganisms with epithelial cells was emphasized. Dysbiosis results in disorder of SCFAs production, and can subsequently affect the integrity of the epithelial barrier. In this review, the mechanisms involved in the loss of the stability of the intestinal barrier, and dysbiosis, which are the reasons for unrequired inflammatory response that can promote the development of IBD in humans, were discussed. However, epithelial cells also coordinate the development and maturation of downstream innate and adaptive immune responses, induced in *lamina propria* and MLNs residing immune cells, resulting in local and/or systemic protective immunity against invading infectious agents.

## Abbreviations

A/E	attaching and effacing
AGR2	anterior gradient 2
AIM	absence in melanoma
AJs	adherens junctions
ALRs	absence in melanoma-like receptors
AMP	adenosinmonophosphate
AMPK	AMP-activated protein kinase
AMPs	antimicrobial peptides
APCs	antigen-presenting cells
CaMKK2	calcium/calmodulin-dependent kinase 2
CAZymes	carbohydrate-active enzymes
CD	Crohn’s disease
ClCa1	calcium-activated chloride channel 1
CLR	C-type lectin receptors
Convr	conventionally raised
CR2	cryptdin-2
CRS	cryptdin related sequence
DAMPs	damage-associated molecular patterns
DCs	dendritic cells
Dll	Delta-like
DSCs	desmocollin types
DSGs	desmoglein types
DSS	dextran-sulfate sodium
EGF	epidermal growth factor
EHEC	enterohaemorrhagic *Escherichia coli*
Emc3	endoplasmic reticulum membrane complex subunit 3
EPEC	enteropathogenic *Escherichia coli*
ER	endoplasmic reticulum
ERK	extracellular signal-regulated kinase
FAE	follicle-associated epithelium
Fc	crystallizable fragment
FCGBP	Fc-globulin binding protein
GALT	gut-associated lymphoid tissue
GAPs	goblet cells-associated antigen passages
GCs	goblet cells
GF	germ-free
GI	gastrointestinal
GP2	Glycoprotein 2
GVHD	graft-versus-host disease
HD5	human α-defensin5
IBD	inflammatory bowel diseases
IBDU	inflammatory bowel diseases-unclassified
icGCs	intercrypt goblet cells
IECs	intestinal epithelial cells
ISCs	intestinal stem cells
JAMs	junctional adhesion molecules
JNK	c-Jun N-terminal kinase
Lgr5^+^	leucine-rich repeat containing G protein-coupled receptor 5
LPS	lipopolysaccharide
Lypd8	Ly6/Plaur domain-containing 8
M	microfold
MAMPs	microbial-associated molecular patterns
MAPK	mitogen-activated protein kinase
MMP	metalloproteinase
MUC	mucin
MyD88	myeloid differentiation primary response gene-88
NEC	necrotizing enterocolitis
NLRs	nucleotide-binding oligomerization domain-like receptors
NOD	nucleotide-binding oligomerization domain
pIgR	polymeric immunoglobulin receptor
PKC	protein kinase C
PRRs	pattern recognition receptors
RegIII	regenerating islet-derived protein 3
RELMβ	resistin-like molecule β
RIG-I	retinoic acid-inducible gene I
RLRs	retinoic acid-inducible gene I-receptors
SCFAs	short-chain fatty acids
SED	subepithelial dome
senGCs	sentinel goblet cells
sIgA	secretory immunoglobulin A
sPLA2	secretory phospholipase A2
T3SS	type III secretion system
Tff3	trefoil factor family peptide 3
Tir	translocated intimin receptor
TIR	Toll/interleukin-1 receptor
TJs	tight junctions
TLRs	Toll-like receptors
TRIF	Toll/interleukin-1 receptor-domain–containing adapter-inducing interferon beta
UC	ulcerative colitis
ZG16	zymogen granule membrane protein 16
ZO	zonula occludens

## References

[1] Kim S.-H., Lee K.-Y., Jang Y.-S. (2012). Mucosal Immune System and M Cell-targeting Strategies for Oral Mucosal Vaccination. Immune Netw..

[2] Sansonetti P.J. (2004). War and peace at mucosal surfaces. Nat. Rev. Immunol..

[3] Burgueño J.F., Abreu M.T. (2020). Epithelial Toll-like receptors and their role in gut homeostasis and disease. Nat. Rev. Gastroenterol. Hepatol..

[4] Kamdar K., Nguyen V., DePaolo R.W. (2013). Toll-like receptor signaling and regulation of intestinal immunity. Virulence.

[5] Schlegel N., Boerner K., Waschke J. (2021). Targeting desmosomal adhesion and signalling for intestinal barrier stabilization in inflammatory bowel diseases—Lessons from experimental models and patients. Acta Physiol..

[6] Molodecky N.A., Soon I.S., Rabi D.M., Ghali W.A., Ferris M., Chernoff G., Benchimol E.I., Panaccione R., Ghosh S., Barkema H.W. (2012). Increasing incidence and prevalence of the inflammatory bowel diseases with time, based on systematic review. Gastroenterology.

[7] Abraham C., Cho J.H. (2009). Inflammatory Bowel Disease. N. Engl. J. Med..

[8] Nishida A., Inoue R., Inatomi O., Bamba S., Naito Y., Andoh A. (2018). Gut microbiota in the pathogenesis of inflammatory bowel disease. Clin. J. Gastroenterol..

[9] Kalinowska-Gacek E., Gierynska M. (2009). Błony śluzowe-stan gotowości immunologicznej. Część I. Życie Weter..

[10] Kong S., Zhang Y.H., Zhang W. (2018). Regulation of intestinal epithelial cells properties and functions by amino acids. BioMed Res. Int..

[11] Karam S.M. (1999). Lineage commitment and maturation of epithelial cells in the gut. Bioscience.

[12] Zhu G., Hu J., Xi R. (2021). The cellular niche for intestinal stem cells: A team effort. Cell Regen..

[13] Gassler N. (2017). Paneth cells in intestinal physiology and pathophysiology. World J. Gastrointest. Pathophysiol..

[14] Allaire J.M., Crowley S.M., Law H.T., Chang S.Y., Ko H.J., Vallance B.A. (2018). The Intestinal Epithelium: Central Coordinator of Mucosal Immunity. Trends Immunol..

[15] Johansson M.E.V., Sjövall H., Hansson G.C. (2013). The gastrointestinal mucus system in health and disease. Nat. Rev. Gastroenterol. Hepatol..

[16] Banerjee A., McKinley E.T., Von Moltke J., Coffey R.J., Lau K.S. (2018). Interpreting heterogeneity in intestinal tuft cell structure and function. J. Clin. Investig..

[17] Mabbott N.A., Donaldson D.S., Ohno H., Williams I.R., Mahajan A. (2013). Microfold (M) cells: Important immunosurveillance posts in the intestinal epithelium. Mucosal Immunol..

[18] Dillon A., Lo D.D. (2019). M cells: Intelligent engineering of mucosal immune surveillance. Front. Immunol..

[19] Kobayashi N., Takahashi D., Takano S., Kimura S., Hase K. (2019). The Roles of Peyer’s Patches and Microfold Cells in the Gut Immune System: Relevance to Autoimmune Diseases. Front. Immunol..

[20] Pelaseyed T., Bergström J.H., Gustafsson J.K., Ermund A., Birchenough G.M.H., Schütte A., van der Post S., Svensson F., Rodríguez-Piñeiro A.M., Nyström E.E.L. (2014). The mucus and mucins of the goblet cells and enterocytes provide the first defense line of the gastrointestinal tract and interact with the immune system. Immunol. Rev..

[21] Bain C.C., Scott C.L., Uronen-Hansson H., Gudjonsson S., Jansson O., Grip O., Guilliams M., Malissen B., Agace W.W., Mowat A.M.I. (2013). Resident and pro-inflammatory macrophages in the colon represent alternative context-dependent fates of the same Ly6C hi monocyte precursors. Mucosal Immunol..

[22] Batlle E., Massagué J. (2019). Transforming Growth Factor-β Signaling in Immunity and Cancer. Immunity.

[23] Mörbe U.M., Jørgensen P.B., Fenton T.M., von Burg N., Riis L.B., Spencer J., Agace W.W. (2021). Human gut-associated lymphoid tissues (GALT); diversity, structure, and function. Mucosal Immunol..

[24] Paone P., Cani P.D. (2020). Mucus barrier, mucins and gut microbiota: The expected slimy partners?. Gut.

[25] Sun W.W., Krystofiak E.S., Leo-Macias A., Cui R., Sesso A., Weigert R., Ebrahim S., Kachar B. (2020). Nanoarchitecture and dynamics of the mouse enteric glycocalyx examined by freeze-etching electron tomography and intravital microscopy. Commun. Biol..

[26] Thornton D.J., Khan N., Mehrotra R., Howard M., Veerman E., Packer N.H., Sheehan J.K. (1999). Salivary mucin MG1 is comprised almost entirely of different glycosylated forms of the MUC5B gene product. Glycobiology.

[27] Khan S.H., Aguirre A., Bobek L.A. (1998). In-situ hybridization localized MUC7 mucin gene expression to the mucous acinar cells of human and MUC7-transgenic mouse salivary glands. Glycoconj. J..

[28] Herath M., Hosie S., Bornstein J.C., Franks A.E., Hill-Yardin E.L. (2020). The Role of the Gastrointestinal Mucus System in Intestinal Homeostasis: Implications for Neurological Disorders. Front. Cell. Infect. Microbiol..

[29] Ambort D., Johansson M.E.V., Gustafsson J.K., Nilsson H.E., Ermund A., Johansson B.R., Koeck P.J.B., Hebert H., Hansson G.C. (2012). Calcium and pH-dependent packing and release of the gel-forming MUC2 mucin. Proc. Natl. Acad. Sci. USA.

[30] Round A.N., Rigby N.M., Garcia De La Torre A., MacIerzanka A., Mills E.N.C., MacKie A.R. (2012). Lamellar structures of MUC2-rich mucin: A potential role in governing the barrier and lubricating functions of intestinal mucus. Biomacromolecules.

[31] Schroeder B.O. (2019). Fight them or feed them: How the intestinal mucus layer manages the gut microbiota. Gastroenterol. Rep..

[32] Nyström E.E.L., Martinez-Abad B., Arike L., Birchenough G.M.H., Nonnecke E.B., Castillo P.A., Svensson F., Bevins C.L., Hansson G.C., Johansson M.E.V. (2021). An intercrypt subpopulation of goblet cells is essential for colonic mucus barrier function. Science.

[33] Schütte A., Ermund A., Becker-Pauly C., Johansson M.E.V., Rodriguez-Pineiro A.M., Bäckhed F., Müller S., Lottaz D., Bond J.S., Hansson G.C. (2014). Microbial-induced meprin β cleavage in MUC2 mucin and a functional CFTR channel are required to release anchored small intestinal mucus. Proc. Natl. Acad. Sci. USA.

[34] Johansson M.E.V., Holmén Larsson J.M., Hansson G.C. (2011). The two mucus layers of colon are organized by the MUC2 mucin, whereas the outer layer is a legislator of host-microbial interactions. Proc. Natl. Acad. Sci. USA.

[35] Birchenough G.M.H., Johansson M.E.V., Gustafsson J.K., Bergström J.H., Hansson G.C. (2015). New developments in goblet cell mucus secretion and function. Mucosal Immunol..

[36] Okumura R., Takeda K. (2017). Roles of intestinal epithelial cells in the maintenance of gut homeostasis. Exp. Mol. Med..

[37] Johansson M.E.V., Phillipson M., Petersson J., Velcich A., Holm L., Hansson G.C. (2008). The inner of the two Muc2 mucin-dependent mucus layers in colon is devoid of bacteria. Proc. Natl. Acad. Sci. USA.

[38] Etienne-Mesmin L., Chassaing B., Desvaux M., De Paepe K., Gresse R., Sauvaitre T., Forano E., van de Wiele T., Schüller S., Juge N. (2019). Experimental models to study intestinal microbes–mucus interactions in health and disease. FEMS Microbiol. Rev..

[39] Nyström E.E.L., Birchenough G.M.H., van der Post S., Arike L., Gruber A.D., Hansson G.C., Johansson M.E.V. (2018). Calcium-activated Chloride Channel Regulator 1 (CLCA1) Controls Mucus Expansion in Colon by Proteolytic Activity. EBioMedicine.

[40] Noah T.K., Kazanjian A., Whitsett J., Shroyer N.F. (2010). SAM pointed domain ETS factor (SPDEF) regulates terminal differentiation and maturation of intestinal goblet cells. Exp. Cell Res..

[41] van Es J.H., Jay P., Gregorieff A., van Gijn M.E., Jonkheer S., Hatzis P., Thiele A., van den Born M., Begthel H., Brabletz T. (2005). Wnt signalling induces maturation of Paneth cells in intestinal crypts. Nat. Cell Biol..

[42] Van Der Flier L.G., Clevers H. (2009). Stem cells, self-renewal, and differentiation in the intestinal epithelium. Annu. Rev. Physiol..

[43] Gregorieff A., Stange D.E., Kujala P., Begthel H., van den Born M., Korving J., Peters P.J., Clevers H. (2009). The Ets-Domain Transcription Factor Spdef Promotes Maturation of Goblet and Paneth Cells in the Intestinal Epithelium. Gastroenterology.

[44] Specian R.D., Oliver M.G. (1991). Functional biology of intestinal goblet cells. Am. J. Physiol.-Cell Physiol..

[45] Dürer U., Hartig R., Bang S., Thim L., Hoffmann W. (2007). TFF3 and EGF induce different migration patterns of intestinal epithelial cells in vitro and trigger increased internalization of E-cadherin. Cell. Physiol. Biochem..

[46] Birchenough G.M.H., Nystrom E.E.L., Johansson M.E.V., Hansson G.C. (2016). A sentinel goblet cell guards the colonic crypt by triggering Nlrp6-dependent Muc2 secretion. Science.

[47] McDole J.R., Wheeler L.W., McDonald K.G., Wang B., Konjufca V., Knoop K.A., Newberry R.D., Miller M.J. (2012). Goblet cells deliver luminal antigen to CD103 + dendritic cells in the small intestine. Nature.

[48] Turula H., Wobus C.E. (2018). The role of the polymeric immunoglobulin receptor and secretory immunoglobulins during mucosal infection and immunity. Viruses.

[49] Grondin J.A., Kwon Y.H., Far P.M., Haq S., Khan W.I. (2020). Mucins in Intestinal Mucosal Defense and Inflammation: Learning From Clinical and Experimental Studies. Front. Immunol..

[50] Chelakkot C., Ghim J., Ryu S.H. (2018). Mechanisms regulating intestinal barrier integrity and its pathological implications. Exp. Mol. Med..

[51] Suzuki T. (2013). Regulation of intestinal epithelial permeability by tight junctions. Cell. Mol. Life Sci..

[52] Bhat A.A., Uppada S., Achkar I.W., Hashem S., Yadav S.K., Shanmugakonar M., Al-Naemi H.A., Haris M., Uddin S. (2019). Tight junction proteins and signaling pathways in cancer and inflammation: A functional crosstalk. Front. Physiol..

[53] Meng W., Takeichi M. (2009). Adherens junction: Molecular architecture and regulation. Cold Spring Harb. Perspect. Biol..

[54] Brown R.C., Davis T.P. (2002). Calcium modulation of adherens and tight junction function: A potential mechanism for blood-brain barrier disruption after stroke. Stroke.

[55] Knights A.J., Funnell A.P.W., Crossley M., Pearson R.C.M. (2012). Holding Tight: Cell Junctions and Cancer Spread. Trends Cancer Res..

[56] Larue L., Ohsugi M., Hirchenhain J., Kemler R. (1994). E-cadherin null mutant embryos fail to form a trophectoderm epithelium. Proc. Natl. Acad. Sci. USA.

[57] Buckley A., Turner J.R. (2018). Cell biology of tight junction barrier regulation and mucosal disease. Cold Spring Harb. Perspect. Biol..

[58] Hermiston M.L., Gordon J.I. (1995). *In vivo* analysis of cadherin function in the mouse intestinal epithelium: Essential roles in adhesion, maintenance of differentiation, and regulation of programmed cell death. J. Cell Biol..

[59] Hermiston M.L., Wong M.H., Gordon J.I. (1996). Forced expression of E-cadherin in the mouse intestinal epithelium slows cell migration and provides evidence for nonautonomous regulation of cell fate in a self-renewing system. Genes Dev..

[60] Schneider M.R., Dahlhoff M., Horst D., Hirschi B., Trülzsch K., Müller-Höcker J., Vogelmann R., Allgäuer M., Gerhard M., Steininger S. (2010). A key role for E-cadherin in intestinal homeostasis and paneth cell maturation. PLoS ONE.

[61] Grill J.I., Neumann J., Hiltwein F., Kolligs F.T., Schneider M.R. (2015). Intestinal E-cadherin Deficiency Aggravates Dextran Sodium Sulfate-Induced Colitis. Dig. Dis. Sci..

[62] Holthöfer B., Windoffer R., Troyanovsky S., Leube R.E. (2007). Structure and Function of Desmosomes. Int. Rev. Cytol..

[63] Gross A., Pack L.A.P., Schacht G.M., Kant S., Ungewiss H., Meir M., Schlegel N., Preisinger C., Boor P., Guldiken N. (2018). Desmoglein 2, but not desmocollin 2, protects intestinal epithelia from injury. Mucosal Immunol..

[64] Kowalczyk A.P., Green K.J. (2013). Structure, function, and regulation of desmosomes. Progress in Molecular Biology and Translational Science.

[65] Kolegraff K., Nava P., Helms M.N., Parkos C.A., Nusrat A. (2011). Loss of desmocollin-2 confers a tumorigenic phenotype to colonic epithelial cells through activation of Akt/β-catenin signaling. Mol. Biol. Cell.

[66] Zihni C., Mills C., Matter K., Balda M.S. (2016). Tight junctions: From simple barriers to multifunctional molecular gates. Nat. Rev. Mol. Cell Biol..

[67] Paradis T., Bègue H., Basmaciyan L., Dalle F., Bon F. (2021). Tight junctions as a key for pathogens invasion in intestinal epithelial cells. Int. J. Mol. Sci..

[68] González-Mariscal L., Domínguez-Calderón A., Raya-Sandino A., Ortega-Olvera J.M., Vargas-Sierra O., Martínez-Revollar G. (2014). Tight junctions and the regulation of gene expression. Semin. Cell Dev. Biol..

[69] Balda M.S., Matter K. (2009). Tight junctions and the regulation of gene expression. Biochim. Biophys. Acta-Biomembr..

[70] Lee B., Moon K.M., Kim C.Y. (2018). Tight junction in the intestinal epithelium: Its association with diseases and regulation by phytochemicals. J. Immunol. Res..

[71] Marchiando A.M., Shen L., Vallen Graham W., Weber C.R., Schwarz B.T., Austin J.R., Raleigh D.R., Guan Y., Watson A.J.M., Montrose M.H. (2010). Caveolin-1-dependent occludin endocytosis is required for TNF-induced tight junction regulation in vivo. J. Cell Biol..

[72] Landy J., Ronde E., English N., Clark S.K., Hart A.L., Knight S.C., Ciclitira P.J., Al-Hassi H.O. (2016). Tight junctions in inflammatory bowel diseases and inflammatory bowel disease associated colorectal cancer. World J. Gastroenterol..

[73] Bazzoni G., Dejana E. (2004). Endothelial cell-to-cell junctions: Molecular organization and role in vascular homeostasis. Physiol. Rev..

[74] Saitou M., Furuse M., Sasaki H., Schulzke J.D., Fromm M., Takano H., Noda T., Tsukita S. (2000). Complex phenotype of mice lacking occludin, a component of tight junction strands. Mol. Biol. Cell.

[75] Lee S.H. (2015). Intestinal Permeability Regulation by Tight Junction: Implication on Inflammatory Bowel Diseases. Intest. Res..

[76] Van Itallie C.M., Anderson J.M. (2006). Claudins and epithelial paracellular transport. Annu. Rev. Physiol..

[77] Furuse M., Hata M., Furuse K., Yoshida Y., Haratake A., Sugitani Y., Noda T., Kubo A., Tsukita S. (2002). Claudin-based tight junctions are crucial for the mammalian epidermal barrier: A lesson from claudin-1-deficient mice. J. Cell Biol..

[78] Zeissig S., Bürgel N., Günzel D., Richter J., Mankertz J., Wahnschaffe U., Kroesen A.J., Zeitz M., Fromm M., Schulzke J.D. (2007). Changes in expression and distribution of claudin 2, 5 and 8 lead to discontinuous tight junctions and barrier dysfunction in active Crohn’s disease. Gut.

[79] Lueschow S.R., McElroy S.J. (2020). The Paneth Cell: The Curator and Defender of the Immature Small Intestine. Front. Immunol..

[80] Van Es J.H., Clevers H. (2014). Paneth cells. Curr. Biol..

[81] Kurashima Y., Tokuhara D., Kamioka M., Inagaki Y., Kiyono H. (2019). Intrinsic control of surface immune and epithelial homeostasis by tissue-resident gut stromal cells. Front. Immunol..

[82] Klockars M., Reitamo S. (1975). Tissue distribution of lysozyme in man. J. Histochem. Cytochem..

[83] Qu X.D., Kent Lloyd K.C., Walsh J.H., Lehrer R.I. (1996). Secretion of type II phospholipase A2 and cryptdin by rat small intestinal Paneth cells. Infect. Immun..

[84] Peterson L.W., Artis D. (2014). Intestinal epithelial cells: Regulators of barrier function and immune homeostasis. Nat. Rev. Immunol..

[85] Mowat A.M., Agace W.W. (2014). Regional specialization within the intestinal immune system. Nat. Rev. Immunol..

[86] Vandamme D., Landuyt B., Luyten W., Schoofs L. (2012). A comprehensive summary of LL-37, the factoctum human cathelicidin peptide. Cell. Immunol..

[87] Barker N., Van Es J.H., Kuipers J., Kujala P., Van Den Born M., Cozijnsen M., Haegebarth A., Korving J., Begthel H., Peters P.J. (2007). Identification of stem cells in small intestine and colon by marker gene Lgr5. Nature.

[88] Propheter D.C., Chara A.L., Harris T.A., Ruhn K.A., Hooper L.V. (2017). Resistin-like molecule β is a bactericidal protein that promotes spatial segregation of the microbiota and the colonic epithelium. Proc. Natl. Acad. Sci. USA.

[89] Ghosh D., Porter E., Shen B., Lee S.K., Wilk D., Drazba J., Yadav S.P., Crabb J.W., Ganz T., Bevins C.L. (2002). Paneth cell trypsin is the processing enzyme for human defensin-5. Nat. Immunol..

[90] Zasloff M. (2002). Trypsin, for the defense. Nat. Immunol..

[91] Yokoi Y., Nakamura K., Yoneda T., Kikuchi M., Sugimoto R., Shimizu Y., Ayabe T. (2019). Paneth cell granule dynamics on secretory responses to bacterial stimuli in enteroids. Sci. Rep..

[92] Ehmann D., Wendler J., Koeninger L., Larsen I.S., Klag T., Berger J., Marette A., Schaller M., Stange E.F., Malek N.P. (2019). Paneth cell α-defensins HD-5 and HD-6 display differential degradation into active antimicrobial fragments. Proc. Natl. Acad. Sci. USA.

[93] Huang M., Yang L., Jiang N., Dai Q., Li R., Zhou Z., Zhao B., Lin X. (2021). Emc3 maintains intestinal homeostasis by preserving secretory lineages. Mucosal Immunol..

[94] Sato T., Van Es J.H., Snippert H.J., Stange D.E., Vries R.G., Van Den Born M., Barker N., Shroyer N.F., Van De Wetering M., Clevers H. (2011). Paneth cells constitute the niche for Lgr5 stem cells in intestinal crypts. Nature.

[95] Vaishnava S., Behrendt C.L., Ismail A.S., Eckmann L., Hooper L.V. (2008). Paneth cells directly sense gut commensals and maintain homeostasis at the intestinal host-microbial interface. Proc. Natl. Acad. Sci. USA.

[96] Price A.E., Shamardani K., Lugo K.A., Deguine J., Roberts A.W., Lee B.L., Barton G.M. (2018). A Map of Toll-like Receptor Expression in the Intestinal Epithelium Reveals Distinct Spatial, Cell Type-Specific, and Temporal Patterns. Immunity.

[97] Wyllie D.H., Kiss-Toth E., Visintin A., Smith S.C., Boussouf S., Segal D.M., Duff G.W., Dower S.K. (2000). Evidence for an Accessory Protein Function for Toll-Like Receptor 1 in Anti-Bacterial Responses. J. Immunol..

[98] Lorenz E., Patel D.D., Hartung T., Schwartz D.A. (2002). Toll-like receptor 4 (TLR4)-deficient murine macrophage cell line as an in vitro assay system to show TLR4-independent signaling of Bacteroides fragilis lipopolysaccharide. Infect. Immun..

[99] Satoh Y. (1988). Atropine inhibits the degranulation of Paneth cells in ex-germ-free mice. Cell Tissue Res..

[100] Stockinger S., Albers T., Duerr C.U., Ménard S., Pütsep K., Andersson M., Hornef M.W. (2014). Interleukin-13-mediated paneth cell degranulation and antimicrobial peptide release. J. Innate Immun..

[101] Farin H.F., Karthaus W.R., Kujala P., Rakhshandehroo M., Schwank G., Vries R.G.J., Kalkhoven E., Nieuwenhuis E.E.S., Clevers H. (2014). Paneth cell extrusion and release of antimicrobial products is directly controlled by immune cell-derived IFN-γ. J. Exp. Med..

[102] Raetz M., Hwang S.H., Wilhelm C.L., Kirkland D., Benson A., Sturge C.R., Mirpuri J., Vaishnava S., Hou B., Defranco A.L. (2013). Parasite-induced T H 1 cells and intestinal dysbiosis cooperate in IFN-γ-dependent elimination of Paneth cells. Nat. Immunol..

[103] Chan J.M., Bhinder G., Sham H.P., Ryz N., Huang T., Bergstrom K.S., Vallance B.A. (2013). CD4+ T cells drive goblet cell depletion during *Citrobacter rodentium* infection. Infect. Immun..

[104] Eriguchi Y., Nakamura K., Yokoi Y., Sugimoto R., Takahashi S., Hashimoto D., Teshima T., Ayabe T., Selsted M.E., Ouellette A.J. (2018). Essential role of IFN-γ in T cell-associated intestinal inflammation. JCI Insight.

[105] Mastroianni J.R., Ouellette A.J. (2009). α-Defensins in enteric innate immunity. Functional paneth cell α-defensins in mouse colonic lumen. J. Biol. Chem..

[106] Mastroianni J.R., Costales J.K., Zaksheske J., Selsted M.E., Salzman N.H., Ouellette A.J. (2012). Alternative luminal activation mechanisms for paneth cell α-defensins. J. Biol. Chem..

[107] Kaser A., Lee A.H., Franke A., Glickman J.N., Zeissig S., Tilg H., Nieuwenhuis E.E.S., Higgins D.E., Schreiber S., Glimcher L.H. (2008). XBP1 Links ER Stress to Intestinal Inflammation and Confers Genetic Risk for Human Inflammatory Bowel Disease. Cell.

[108] Sidiq T., Yoshihama S., Downs I., Kobayashi K.S. (2016). Nod2: A critical regulator of ileal microbiota and Crohn’s disease. Front. Immunol..

[109] Koslowski M.J., Kübler I., Chamaillard M., Schaeffeler E., Reinisch W., Wang G., Beisner J., Teml A., Peyrin-Biroulet L., Winter S. (2009). Genetic variants of Wnt transcription factor TCF-4 (TCF7L2) putative promoter region are associated with small intestinal Crohn’s disease. PLoS ONE.

[110] Hodin C.M., Verdam F.J., Grootjans J., Rensen S.S., Verheyen F.K., Dejong C.H.C., Buurman W.A., Greve J.W., Lenaerts K. (2011). Reduced Paneth cell antimicrobial protein levels correlate with activation of the unfolded protein response in the gut of obese individuals. J. Pathol..

[111] Hayase E., Hashimoto D., Nakamura K., Noizat C., Ogasawara R., Takahashi S., Ohigashi H., Yokoi Y., Sugimoto R., Matsuoka S. (2017). R-Spondin1 expands Paneth cells and prevents dysbiosis induced by graft-versus-host disease. J. Exp. Med..

[112] Eriguchi Y., Takashima S., Oka H., Shimoji S., Nakamura K., Uryu H., Shimoda S., Iwasaki H., Shimono N., Ayabe T. (2012). Graft-versus-host disease disrupts intestinal microbial ecology by inhibiting Paneth cell production of α-defensins. Blood.

[113] Elphick D.A., Mahida Y.R. (2005). Paneth cells: Their role in innate immunity and inflammatory disease. Gut.

[114] Smith V.C., Genta R.M. (2000). Role of Helicobacter pylori gastritis in gastric atrophy, intestinal metaplasia, and gastric neoplasia. Microsc. Res. Tech..

[115] Singh R., Balasubramanian I., Zhang L., Gao N. (2020). Metaplastic Paneth Cells in Extra-Intestinal Mucosal Niche Indicate a Link to Microbiome and Inflammation. Front. Physiol..

[116] Mei X., Gu M., Li M. (2020). Plasticity of Paneth cells and their ability to regulate intestinal stem cells. Stem Cell Res. Ther..

[117] Sato T., Vries R.G., Snippert H.J., Van De Wetering M., Barker N., Stange D.E., Van Es J.H., Abo A., Kujala P., Peters P.J. (2009). Single Lgr5 stem cells build crypt-villus structures in vitro without a mesenchymal niche. Nature.

[118] Laederich M.B., Funes-Duran M., Yen L., Ingalla E., Wu X., Carraway K.L., Sweeney C. (2004). The leucine-rich repeat protein LRIG1 is a negative regulator of ErbB family receptor tyrosine kinases. J. Biol. Chem..

[119] VanDussen K.L., Carulli A.J., Keeley T.M., Patel S.R., Puthoff B.J., Magness S.T., Tran I.T., Maillard I., Siebel C., Kolterud Å. (2012). Notch signaling modulates proliferation and differentiation of intestinal crypt base columnar stem cells. Development.

[120] Poulsen S.S., Nexø E., Skov Olsen P., Hess J., Kirkegaard P. (1986). Immunohistochemical localization of epidermal growth factor in rat and man. Histochemistry.

[121] Rizk P., Barker N. (2012). Gut stem cells in tissue renewal and disease: Methods, markers, and myths. Wiley Interdiscip. Rev. Syst. Biol. Med..

[122] Bastide P., Darido C., Pannequin J., Kist R., Robine S., Marty-Double C., Bibeau F., Scherer G., Joubert D., Hollande F. (2007). Sox9 regulates cell proliferation and is required for Paneth cell differentiation in the intestinal epithelium. J. Cell Biol..

[123] Mori-Akiyama Y., van den Born M., van Es J.H., Hamilton S.R., Adams H.P., Zhang J., Clevers H., de Crombrugghe B. (2007). SOX9 Is Required for the Differentiation of Paneth Cells in the Intestinal Epithelium. Gastroenterology.

[124] Schmitt M., Schewe M., Sacchetti A., Feijtel D., van de Geer W.S., Teeuwssen M., Sleddens H.F., Joosten R., van Royen M.E., van de Werken H.J.G. (2018). Paneth Cells Respond to Inflammation and Contribute to Tissue Regeneration by Acquiring Stem-like Features through SCF/c-Kit Signaling. Cell Rep..

[125] Yu S., Tong K., Zhao Y., Balasubramanian I., Yap G.S., Ferraris R.P., Bonder E.M., Verzi M.P., Gao N. (2018). Paneth Cell Multipotency Induced by Notch Activation following Injury. Cell Stem Cell.

[126] Yan K.S., Gevaert O., Zheng G.X.Y., Anchang B., Probert C.S., Larkin K.A., Davies P.S., Cheng Z.F., Kaddis J.S., Han A. (2017). Intestinal Enteroendocrine Lineage Cells Possess Homeostatic and Injury-Inducible Stem Cell Activity. Cell Stem Cell.

[127] Cadwell K., Liu J.Y., Brown S.L., Miyoshi H., Loh J., Lennerz J.K., Kishi C., Kc W., Carrero J.A., Hunt S. (2008). A key role for autophagy and the autophagy gene Atg16l1 in mouse and human intestinal Paneth cells. Nature.

[128] Wehkamp J., Salzman N.H., Porter E., Nuding S., Weichenthal M., Petras R.E., Shen B., Schaeffeler E., Schwab M., Linzmeier R. (2005). Reduced Paneth cell α-defensins in ileal Crohn’s disease. Proc. Natl. Acad. Sci. USA.

[129] He D., Wu H., Xiang J., Ruan X., Peng P., Ruan Y., Chen Y.G., Wang Y., Yu Q., Zhang H. (2020). Gut stem cell aging is driven by mTORC1 via a p38 MAPK-p53 pathway. Nat. Commun..

[130] Nalapareddy K., Nattamai K.J., Kumar R.S., Karns R., Wikenheiser-Brokamp K.A., Sampson L.L., Mahe M.M., Sundaram N., Yacyshyn M.B., Yacyshyn B. (2017). Canonical Wnt Signaling Ameliorates Aging of Intestinal Stem Cells. Cell Rep..

[131] Pinto D., Gregorieff A., Begthel H., Clevers H. (2003). Canonical Wnt signals are essential for homeostasis of the intestinal epithelium. Genes Dev..

[132] Pentinmikko N., Iqbal S., Mana M., Andersson S., Cognetta A.B., Suciu R.M., Roper J., Luopajärvi K., Markelin E., Gopalakrishnan S. (2019). Notum produced by Paneth cells attenuates regeneration of aged intestinal epithelium. Nature.

[133] Kakugawa S., Langton P.F., Zebisch M., Howell S.A., Chang T.H., Liu Y., Feizi T., Bineva G., O’Reilly N., Snijders A.P. (2015). Notum deacylates Wnt proteins to suppress signalling activity. Nature.

[134] Zhu L., Han J., Li L., Wang Y., Li Y., Zhang S. (2019). Claudin family participates in the pathogenesis of inflammatory bowel diseases and colitis-associated colorectal cancer. Front. Immunol..

[135] Hansson G.C. (2019). Mucus and mucins in diseases of the intestinal and respiratory tracts. J. Intern. Med..

[136] Cone R.A. (2009). Barrier properties of mucus. Adv. Drug Deliv. Rev..

[137] Lidell M.E., Johansson M.E.V., Mörgelin M., Asker N., Gum J.R., Kim Y.S., Hansson G.C. (2003). The recombinant C-terminus of the human MUC2 mucin forms dimers in Chinese-hamster ovary cells and heterodimers with full-length MUC2 in LS 174T cells. Biochem. J..

[138] Abreu M.T. (2010). Toll-like receptor signalling in the intestinal epithelium: How bacterial recognition shapes intestinal function. Nat. Rev. Immunol..

[139] McClure R., Massari P. (2014). TLR-dependent human mucosal epithelial cell responses to microbial pathogens. Front. Immunol..

[140] McKernan D.P. (2019). Toll-like receptors and immune cell crosstalk in the intestinal epithelium. AIMS Allergy Immunol..

[141] Bugge M., Bergstrom B., Eide O.K., Solli H., Kjønstad I.F., Stenvik J., Espevik T., Nilsen N.J. (2017). Surface Toll-like receptor 3 expression in metastatic intestinal epithelial cells induces inflammatory cytokine production and promotes invasiveness. J. Biol. Chem..

[142] Gewirtz A.T., Navas T.A., Lyons S., Godowski P.J., Madara J.L. (2001). Cutting Edge: Bacterial Flagellin Activates Basolaterally Expressed TLR5 to Induce Epithelial Proinflammatory Gene Expression. J. Immunol..

[143] Lee J., Mo J.H., Katakura K., Alkalay I., Rucker A.N., Liu Y.T., Lee H.K., Shen C., Cojocaru G., Shenouda S. (2006). Maintenance of colonic homeostasis by distinctive apical TLR9 signalling in intestinal epithelial cells. Nat. Cell Biol..

[144] Van der Sluis M., De Koning B.A.E., De Bruijn A.C.J.M., Velcich A., Meijerink J.P.P., Van Goudoever J.B., Büller H.A., Dekker J., Van Seuningen I., Renes I.B. (2006). Muc2-Deficient Mice Spontaneously Develop Colitis, Indicating That MUC2 Is Critical for Colonic Protection. Gastroenterology.

[145] Carvalho F.A., Koren O., Goodrich J.K., Johansson M.E.V., Nalbantoglu I., Aitken J.D., Su Y., Chassaing B., Walters W.A., González A. (2012). Transient inability to manage proteobacteria promotes chronic gut inflammation in TLR5-deficient mice. Cell Host Microbe.

[146] Chassaing B., Ley R.E., Gewirtz A.T. (2014). Intestinal epithelial cell toll-like receptor 5 regulates the intestinal microbiota to prevent low-grade inflammation and metabolic syndrome in mice. Gastroenterology.

[147] Kamdar K., Johnson A.M.F., Chac D., Myers K., Kulur V., Truevillian K., DePaolo R.W. (2018). Innate Recognition of the Microbiota by TLR1 Promotes Epithelial Homeostasis and Prevents Chronic Inflammation. J. Immunol..

[148] Choteau L., Vancraeyneste H., Le Roy D., Dubuquoy L., Romani L., Jouault T., Poulain D., Sendid B., Calandra T., Roger T. (2017). Role of TLR1, TLR2 and TLR6 in the modulation of intestinal inflammation and Candida albicans elimination. Gut Pathog..

[149] Xu Y., Wang N., Tan H.Y., Li S., Zhang C., Feng Y. (2020). Function of *Akkermansia muciniphila* in Obesity: Interactions with Lipid Metabolism, Immune Response and Gut Systems. Front. Microbiol..

[150] Plovier H., Everard A., Druart C., Depommier C., Van Hul M., Geurts L., Chilloux J., Ottman N., Duparc T., Lichtenstein L. (2017). A purified membrane protein from *Akkermansia muciniphila* or the pasteurized bacterium improves metabolism in obese and diabetic mice. Nat. Med..

[151] Sommer F., Bäckhed F. (2013). The gut microbiota-masters of host development and physiology. Nat. Rev. Microbiol..

[152] Szentkuti L., Riedesel H., Enss M.-L., Gaertner K., von Engelhardt W. (1990). Pre-epithelial mucus layer in the colon of conventional and germ-free rats. Histochem. J..

[153] Goto Y. (2019). Epithelial cells as a transmitter of signals from commensal bacteria and host immune cells. Front. Immunol..

[154] Bergström A., Kristensen M.B., Bahl M.I., Metzdorff S.B., Fink L.N., Frøkiær H., Licht T.R. (2012). Nature of bacterial colonization influences transcription of mucin genes in mice during the first week of life. BMC Res. Notes.

[155] Petersson J., Schreiber O., Hansson G.C., Gendler S.J., Velcich A., Lundberg J.O., Roos S., Holm L., Phillipson M. (2011). Importance and regulation of the colonic mucus barrier in a mouse model of colitis. Am. J. Physiol.-Gastrointest. Liver Physiol..

[156] Johansson M.E.V., Jakobsson H.E., Holmén-Larsson J., Schütte A., Ermund A., Rodríguez-Piñeiro A.M., Arike L., Wising C., Svensson F., Bäckhed F. (2015). Normalization of host intestinal mucus layers requires long-term microbial colonization. Cell Host Microbe.

[157] Schoenborn A.A., von Furstenberg R.J., Valsaraj S., Hussain F.S., Stein M., Shanahan M.T., Henning S.J., Gulati A.S. (2019). The enteric microbiota regulates jejunal Paneth cell number and function without impacting intestinal stem cells. Gut Microbes.

[158] Moor K., Diard M., Sellin M.E., Felmy B., Wotzka S.Y., Toska A., Bakkeren E., Arnoldini M., Bansept F., Co A.D. (2017). High-avidity IgA protects the intestine by enchaining growing bacteria. Nature.

[159] Ohno H., Hase K. (2010). Glycoprotein 2 (GP2) grabbing the fimH+ bacteria into m cells for mucosal immunity. Gut Microbes.

[160] Hase K., Kawano K., Nochi T., Pontes G.S., Fukuda S., Ebisawa M., Kadokura K., Tobe T., Fujimura Y., Kawano S. (2009). Uptake through glycoprotein 2 of FimH + bacteria by M cells initiates mucosal immune response. Nature.

[161] Moon C., Vandussen K.L., Miyoshi H., Stappenbeck T.S. (2014). Development of a primary mouse intestinal epithelial cell monolayer culture system to evaluate factors that modulate IgA transcytosis. Mucosal Immunol..

[162] Bruno M.E.C., Frantz A.L., Rogier E.W., Johansen F.E., Kaetzel C.S. (2011). Regulation of the polymeric immunoglobulin receptor by the classical and alternative NF-κB pathways in intestinal epithelial cells. Mucosal Immunol..

[163] Okumura R., Kurakawa T., Nakano T., Kayama H., Kinoshita M., Motooka D., Gotoh K., Kimura T., Kamiyama N., Kusu T. (2016). Lypd8 promotes the segregation of flagellated microbiota and colonic epithelia. Nature.

[164] Deng W., Vallance B.A., Li Y., Puente J.L., Finlay B.B. (2003). *Citrobacter rodentium* translocated intimin receptor (Tir) is an essential virulence factor needed for actin condensation, intestinal colonization and colonic hyperplasia in mice. Mol. Microbiol..

[165] Batchelor M., Prasannan S., Daniell S., Reece S., Connerton I., Bloomberg G., Dougan G., Frankel G., Matthews S. (2000). Structural basis for recognition of the translocated intimin receptor (Tir) by intimin from enteropathogenic Escherichia coli. EMBO J..

[166] Okumura R., Kodama T., Hsu C.-C., Sahlgren B.H., Hamano S., Kurakawa T., Iida T., Takeda K. (2020). Lypd8 inhibits attachment of pathogenic bacteria to colonic epithelia. Mucosal Immunol..

[167] Bergström J.H., Birchenough G.M.H., Katona G., Schroeder B.O., Schütte A., Ermund A., Johansson M.E.V., Hansson G.C. (2016). Gram-positive bacteria are held at a distance in the colon mucus by the lectin-like protein ZG16. Proc. Natl. Acad. Sci. USA.

[168] Bhattacharya T., Ghosh T.S., Mande S.S. (2015). Global profiling of carbohydrate active enzymes in human gut microbiome. PLoS ONE.

[169] Venegas D.P., De La Fuente M.K., Landskron G., González M.J., Quera R., Dijkstra G., Harmsen H.J.M., Faber K.N., Hermoso M.A. (2019). Short chain fatty acids (SCFAs)mediated gut epithelial and immune regulation and its relevance for inflammatory bowel diseases. Front. Immunol..

[170] Park J.H., Kotani T., Konno T., Setiawan J., Kitamura Y., Imada S., Usui Y., Hatano N., Shinohara M., Saito Y. (2016). Promotion of intestinal epithelial cell turnover by commensal bacteria: Role of short-chain fatty acids. PLoS ONE.

[171] Zhang J., Yi M., Zha L., Chen S., Li Z., Li C., Gong M., Deng H., Chu X., Chen J. (2016). Sodium Butyrate Induces Endoplasmic Reticulum Stress and Autophagy in Colorectal Cells: Implications for Apoptosis. PLoS ONE.

[172] Kelly C.J., Colgan S.P. (2016). Breathless in the Gut: Implications of Luminal O2 for Microbial Pathogenicity. Cell Host Microbe.

[173] Rivera-Chávez F., Zhang L.F., Faber F., Lopez C.A., Byndloss M.X., Olsan E.E., Xu G., Velazquez E.M., Lebrilla C.B., Winter S.E. (2016). Depletion of Butyrate-Producing Clostridia from the Gut Microbiota Drives an Aerobic Luminal Expansion of *Salmonella*. Cell Host Microbe.

[174] Winter S.E., Winter M.G., Xavier M.N., Thiennimitr P., Poon V., Keestra A.M., Laughlin R.C., Gomez G., Wu J., Lawhon S.D. (2013). Host-derived nitrate boosts growth of E. coli in the inflamed gut. Science.

[175] Wang H.B., Wang P.Y., Wang X., Wan Y.L., Liu Y.C. (2012). Butyrate enhances intestinal epithelial barrier function via up-regulation of tight junction protein claudin-1 transcription. Dig. Dis. Sci..

[176] Rodríguez-Colman M.J., Schewe M., Meerlo M., Stigter E., Gerrits J., Pras-Raves M., Sacchetti A., Hornsveld M., Oost K.C., Snippert H.J. (2017). Interplay between metabolic identities in the intestinal crypt supports stem cell function. Nature.

